# Lnc Tmem235 promotes repair of early steroid-induced osteonecrosis of the femoral head by inhibiting hypoxia-induced apoptosis of BMSCs

**DOI:** 10.1038/s12276-022-00875-0

**Published:** 2022-11-16

**Authors:** Fei Zhang, Wuxun Peng, Tao Wang, Jian Zhang, Wentao Dong, Chuan Wang, Zhihong Xie, Hong Luo, Gang Liu

**Affiliations:** 1 Department of Orthopedics, The Affiliated Hospital of Guizhou Medical University, Guiyang, Guizhou 550004 China; 2 School of Clinical Medicine, Guizhou Medical University, Guiyang, Guizhou 550004 China

**Keywords:** Diseases, Mesenchymal stem cells

## Abstract

Bone marrow mesenchymal stem cells (BMSCs) have been used in the treatment of early steroid-induced osteonecrosis of the femoral head (SONFH). However, the hypoxic microenvironment in the osteonecrotic area leads to hypoxia-induced apoptosis of transplanted BMSCs, which limits their efficacy. Therefore, approaches that inhibit hypoxia-induced apoptosis of BMSCs are promising for augmenting the efficacy of BMSC transplantation. Our present study found that under hypoxia, the expression of the long noncoding RNA (Lnc) transmembrane protein 235 (Tmem235) was downregulated, the expression of Bcl-2-associated X protein was upregulated, the expression of B-cell lymphoma-2 protein was downregulated, and the apoptotic rate of BMSCs was over 70%. However, overexpression of Lnc Tmem235 reversed hypoxia-induced apoptosis of BMSCs and promoted their survival. These results demonstrated that Lnc Tmem235 effectively inhibited hypoxia-induced apoptosis of BMSCs. Mechanistically, we found that Lnc Tmem235 exhibited competitive binding to miR-34a-3p compared with BIRC5 mRNA, which is an inhibitor of apoptosis; this competitive binding relieved the silencing effect of miR-34a-3p on BIRC5 mRNA to ultimately inhibit hypoxia-induced apoptosis of BMSCs by promoting the expression of BIRC5. Furthermore, we cocultured BMSCs overexpressing Lnc Tmem235 with xenogeneic antigen-extracted cancellous bone to construct tissue-engineered bone to repair a model of early SONFH in vivo. The results showed that overexpression of Lnc Tmem235 effectively reduced apoptosis of BMSCs in the hypoxic microenvironment of osteonecrosis and improved the effect of BMSC transplantation. Taken together, our findings show that Lnc Tmem235 inhibited hypoxia-induced apoptosis of BMSCs by regulating the miR-34a-3p/BIRC5 axis, thus improving the transplantation efficacy of BMSCs for treating early SONFH.

## Bone disease: A way to promote survival of transplanted stem cells

Boosting the activity of a regulatory RNA molecule could improve the efficiency of stem cell therapies for a progressive bone disorder that results from prolonged steroid use for other medical conditions. Bone marrow-derived stem cells are commonly used to treat a steroid-induced disease that occurs when cells in the highest part of the thigh bone break down, leading to hip joint dysfunction and chronic pain. Wuxun Peng and colleagues from the Affiliated Hospital of Guizhou Medical University in Guiyang, China, showed that these cells often struggle to survive in the oxygen-poor environment of decaying bone tissue due to changes in the expression levels of various regulatory molecules, one of which is a long non-coding RNA called Tmem235. When experimentally activated, this RNA prevents the stem cells from dying under low-oxygen conditions. A similar strategy may be beneficial in patients.

## Introduction

The incidence of steroid-induced osteonecrosis of the femoral head (SONFH) is increasing year by year and accounts for 25%–51% of the total cases of osteonecrosis of the femoral head, ranking first in nontraumatic osteonecrosis of the femoral head^1,2^. Without timely treatment, the collapse rate of osteonecrosis of the femoral head within two years is more than 80%, and the disability rate is markedly high. Therefore, early treatment is particularly important^2–4^. Studies have shown that tissue-engineered bone with bone marrow mesenchymal stem cells (BMSCs) as the core can promote bone repair, which represents a novel method for treating early SONFH^5–9^. However, SONFH has a unique pathological factor; that is, the oxygen concentration in the necrotic area of the femoral head in SONFH cases is usually lower than 1%, and a local hypoxic microenvironment is formed, which can lead to hypoxia-induced apoptosis of transplanted BMSCs, limiting their capacity for osteogenic repair^10–17^. Therefore, the identification and development of methods for inhibiting hypoxia-induced apoptosis of BMSCs are key to further improving the transplantation effect of BMSCs in early SONFH. At present, methods to inhibit apoptosis of BMSCs mainly include drug pretreatments, improvement of mitochondrial function, clearance of excessive reactive oxygen species (ROS) and inhibition of apoptosis-related proteins; however, the efficacies of these methods remain unsatisfactory^18–21^. Therefore, it is important to identify novel targets and develop new methods for inhibiting hypoxia-induced apoptosis of BMSCs and improving the efficacy of BMSC transplantation.

Long noncoding RNAs (lncRNAs) are noncoding RNA molecules with transcripts longer than 200 nucleotides. Upon their initial discovery, lncRNAs were initially considered noise in the process of gene transcription and were not thought to have any biological function. However, recent studies have shown that lncRNAs can regulate gene expression through DNA methylation, histone modification, control of transcription factors, and regulation of RNA stability and subcellular localization at the pretranscriptional, transcriptional and post-transcriptional levels, thus interfering with a variety of physiological activities, such as apoptosis^22–25^. For example, Lnc TNRC6C-AS1 inhibits apoptosis of thyroid cancer cells by promoting methylation of STK4^26^. Additionally, Lnc TCF7 recruits the transcription factor complex SWI/SNF to the TCF7 promoter region to regulate its expression, thus promoting the self-renewal and proliferation of liver cancer stem cells^27^. Furthermore, Lnc H19 regulates the intracellular localization of HIF-1α at the post-translational level, thus interfering with the apoptosis of smooth muscle cells^28^. In addition, lncRNA-regulated gene expression is closely related to microRNAs (miRNAs)^29,30^.

MiRNAs are small noncoding single-stranded RNAs that can bind to the untranslated region (UTR) at the 3’ end of a target mRNA (mRNA 3’UTR) to inhibit mRNA translation and cause gene silencing. Studies have shown that lncRNAs can act as competitive endogenous RNAs (ceRNAs) to competitively bind miRNAs with the mRNA of the target gene, thereby relieving the silencing effect of miRNAs on the target gene to ultimately promote expression of the target gene and interfere with various physiological activities such as apoptosis^29,30^. For example, Lnc CARL can be used as a sponge of endogenous miR-539 to regulate the expression of PHB2, thereby intervening in cardiomyocyte apoptosis^31^. As another example, Lnc LOXL1-AS1 can be used as a ceRNA to compete with RAP1B mRNA for binding with miR-28-5p to promote RAP1B expression, ultimately inhibiting apoptosis of endometrial cancer cells^32^. However, little is known about the role of lncRNAs and miRNAs in the process of hypoxia-induced apoptosis of BMSCs. Hence, it is not clear whether novel targets or methods can be identified for inhibiting hypoxia-induced apoptosis of BMSCs and whether inhibition of hypoxia-induced apoptosis of BMSCs may further improve the transplantation efficacy of BMSCs for treating early SONFH.

In the present study, we explored the effects and mechanisms of the interaction between Lnc Tmem235 and miR-34a-3p on hypoxia-induced apoptosis of BMSCs. Furthermore, we evaluated the effects of inhibiting hypoxia-induced apoptosis of BMSCs in terms of treating early SONFH. Collectively, our findings may contribute to the identification of novel targets and the development of methods for inhibiting hypoxia-induced apoptosis of BMSCs and improving the efficacy of BMSC transplantation.

## Materials and methods

### Ethical statement

We extracted BMSCs from young male Sprague‒Dawley (SD) rats (20–30 g) and established SONFH models using adult male SD rats (500–600 g). SD rats were provided by the Laboratory Animal Center of Guizhou Medical University (Guiyang, China). All experiments were approved by the Experimental Animal Ethics Committee of Guizhou Medical University, and the experimental facility certificate number is SYXK (Qian) 2018-0001. All procedures were performed in accordance with our Institutional Guidelines for Animal Research, and the investigation conformed to the Guide for the Care and Use of Laboratory Animals published by the US National Institutes of Health (NIH Publication No. 85–23, revised in 1996).

### BMSC cultures

The bilateral femurs and tibia of male SD rats (weighing 20–30 g) were removed, and the bone marrow fluid was obtained by flushing the bone marrow cavity under aseptic conditions. The bone marrow fluid was diluted with phosphate-buffered saline (PBS; HyClone, Logan, UT, USA) at a ratio of 1:1 and was then transferred into a single cell suspension, after which it was centrifuged at 600 g/min for 5 min. The precipitated bone marrow was slowly added to the Percoll separation solution (1.073 g/mL; Pharmacia, NYC, USA) along the tube wall and centrifuged at 900 g/min for 30 min, and nucleated cells were absorbed. We then washed the cells with PBS and centrifuged them at 600 g/min for 15 min. The cells were resuspended in complete L-glutamine Dulbecco’s modified Eagle’s medium (L-DMEM; Gibco, Waltham, MA, USA) containing 10% fetal bovine serum (FBS; Gibco) and 1% double antibiotics (HyClone), after which they were cultured at 37 °C and 5% CO_2_. When the primary BMSC confluence reached approximately 90% of the bottom of the culture bottle, the cells were digested with 0.25% trypsin − 0.02% ethylenediaminetetraacetic acid (EDTA; Gibco) at 37 °C, and the cells were passaged at a 1:3 ratio. Second-generation BMSCs were used for the subsequent experiments.

### Osteogenic differentiation of BMSCs

Second-generation BMSCs were inoculated in six-well plates. When the cell confluence was approximately 60% at the bottom of the culture bottle, according to the instructions of the BMSC osteogenesis induction kit (Cyagen Biosciences, Santa Clara, CA, USA), the medium in the experimental group was replaced with BMSC osteogenesis induction medium, while the cells in the control group remained in complete L-DMEM. After two weeks of osteogenic induction, the cells were fixed, alkaline phosphatase (ALP) (Cyagen Biosciences) staining was used to detect ALP activity, and 0.1% alizarin red (Cyagen Biosciences) staining was used to identify calcium nodules.

### Lipogenic differentiation of BMSCs

Second-generation BMSCs were inoculated in six-well plates. When the cell fusion degree reached 100% or reached overfusion, according to the description of the BMSC adipogenic induction kit (Cyagen Biosciences), the medium of the experimental group was replaced with BMSC adipogenic differentiation medium, while the cells in the control group remained in complete L-DMEM. After three weeks of adipogenic induction, lipid droplets were identified by oil red O staining (Cyagen Biosciences).

### Chondrogenic differentiation of BMSCs

Second-generation BMSCs were inoculated in six-well plates. When the cell fusion degree reached approximately 60%, according to the description of the BMSC chondrogenic induction kit (Cyagen Biosciences), the medium in the experimental group was replaced with BMSC chondrogenic induction medium, while the cells in the control group remained in complete L-DMEM. After four weeks of chondrogenic induction, acidic mucopolysaccharides in cartilage tissue were identified by Alcian blue staining (Cyagen Biosciences).

### Identification of surface antigens of BMSCs

The density of second-generation BMSCs was adjusted to 2 × 10^7^ cells/mL. The control group received 50 µL of buffer, while the single-label group received 5 µL of hamster anti-CD29-AF647 (BD Biosciences, Franklin Lakes, NJ, USA), mouse anti-CD90-PECyTM7 (BD Biosciences), mouse anti-CD106-PE (BD Biosciences), mouse anti-CD11b-V450 (BD Biosciences), or mouse anti-CD45-FITC (BD Biosciences) to each branch-flow sampling tube, after which 45 µL of buffer was added to each tube. The multicolor group received 5 µL of each antibody into a one-branch-flow sampling tube, and then, 25 µL of buffer was added. Next, 50 µL of cell suspension was added to each flow tube, the tubes were incubated at room temperature for 30 min and washed twice with staining buffer, and then, 500 µL of buffer was added to each tube for detection by flow cytometry (Beckman Coulter Life Sciences, Brea, CA, USA).

### Cell hypoxia model

For induction of hypoxia, second-generation BMSCs were continuously treated with mixed gas at an oxygen concentration of 0%, nitrogen concentration of 95%, and carbon dioxide concentration of 5% for 48 h.

### Adenosine triphosphate (ATP)

Second-generation BMSCs were collected into centrifuge tubes. According to the instructions of the ATP content detection kit (Solarbio, Beijing, China), 1 mL of the extracted liquid was added to five million cells, which were crushed by ultrasound for 1 min (ice bath, 200 W, ultrasonic time was 2 s, stopping time was 1 s) and centrifuged at 10000 g/min for 10 min. Then, the supernatant of each sample was collected in an Eppendorf tube and mixed with 500 μL of chloroform prior to centrifugation at 10000 g/min for 3 min. The supernatant was fully mixed with the working solution, and after 10 s, the absorbance value A1 under 340 nm was determined via an enzyme meter (BioTek, Winooski, VT, USA). Then, a 96-well plate was placed into an incubator at 37 °C for 180 s, and the absorbance value A2 at 190 s was determined. The absorbance values of the sample solution and ATP standard solution were determined at the same time, and finally, the ATP content was calculated as follows: ATP content (μmol/10^6^ cells) = 0.125 × ΔA sample ÷ ΔA standard, ΔA sample = A2 sample − A1 sample, ΔA standard = A2 standard − A1 standard.

### Mitochondrial membrane potential

After hypoxic treatment of second-generation BMSCs, the cells were washed with PBS, and the reaction mixture was prepared according to the instructions of the mitochondrial membrane potential detection kit (KeyGEN BioTECH, Nanjing, China). The reaction mixture was added, incubated at 37 °C for 30 min, and washed three times with PBS, and the fluorescence was observed under a laser confocal microscope (Carl Zeiss AG, Oberkochen, Germany).

### ROS content

After hypoxic treatment of second-generation BMSCs, the cells were washed with PBS buffer and stained according to the instructions of the DCFH-DA fluorescence probe kit (Sigma-Aldrich, St. Louis, MO, USA). The cells were incubated in a 5% CO_2_ incubator at 37 °C for 30 min, and green fluorescence was observed by laser confocal microscopy.

### TdT-mediated dUTP nick-end labeling (TUNEL) / 4′,6-diamidino-2-phenylindole (DAPI)

Second-generation BMSCs were fixed with 4% paraformaldehyde (Solarbio) at room temperature for 30 min and permeabilized with 0.3% Triton X-100 (Solarbio) for 6 min. Then, TUNEL detection solution (Beyotime, Shanghai, China) was added and incubated at 37 °C for 60 min in the dark, after which the samples were washed with PBS and stained with DAPI (Solarbio) for 5 min.

### Annexin V-fluorescein isothiocyanate (FITC) / propidium iodide (PI)

Second-generation BMSCs were washed with PBS, and then, 5 μL of Annexin V-FITC and 5 μL of PI were directly added according to the instructions of the Annexin V-FITC apoptosis detection kit (BD Biosciences). The cells were gently vortexed and incubated at room temperature in the dark for 15 min and were then detected by flow cytometry.

### Lentiviral transfections

Lentiviruses were purchased from China Shanghai Genechem Co., Ltd., according to the best MOI (MOI = 80) and the best transfection conditions (Eni.s+Polybrane) found during our transfection preexperiments. Second-generation BMSCs were infected with these lentiviruses, and a blank control and negative control were also established at this time. After 12 h, the culture medium was changed to complete L-DMEM. On the fourth day after infection, the stable strain was screened by adding 2 μg/mL puromycin. When all cells in the blank control group died, the concentration of puromycin was reduced to 1 μg/mL to maintain the screening.

### Microarray and bioinformatic analyses

The total RNA of three pairs of cell samples treated with regular oxygen and hypoxia was extracted by TRIzol reagent (Invitrogen, Carlsbad, CA, USA). The RNA quality was confirmed by formaldehyde agarose gel electrophoresis and quantified by a NanoDrop ND-1000. Double-stranded cDNA was synthesized from total RNA samples without rRNA and was then labeled with cDNA and hybridized to SD rat lncRNA expression microarray v3.0 (8×60 K, Arraystar, Rockville, MD, USA). After the microarray was cleaned, the slides were scanned with an Agilent Microarray Scanner (Agilent p/n G2565BA). Raw data were extracted as paired files using Agilent Feature Extraction. The differentially expressed genes were identified by a random variance model. Paired t tests were used to calculate P values. The thresholds of upregulated and downregulated genes are expressed as fold change (FC) > 2.0 and *P* < 0.05. Hierarchical clustering was performed using clustering software to analyze the expression patterns of lncRNAs and mRNAs. The gene coexpression network between lncRNAs and mRNAs was analyzed by Cytoscape software. GO and KEGG analyses were performed to cluster the differentially expressed mRNAs (FC > 2).

### RNA-fluorescence in situ hybridization (RNA-FISH)

The second-generation BMSCs were inoculated in the laser confocal culture dish, and when the degree of cell fusion reached 60%–70%, the cells were fixed, made transparent and blocked according to the instructions of the RNA-FISH detection kit (RiboBio, Guangzhou, China). Then, LncRNA Fish Probe Mix (RiboBio) was used for light-avoiding hybridization overnight at 37 °C; 18 S and U6 (RiboBio) were used as positive controls for the cytoplasm and nucleus, respectively. The nucleus was labeled with DAPI (RiboBio). PBS was used to wash the cells. Anti-fluorescence quenching agent (Solarbio) was then added, and the fluorescence was observed with a laser confocal microscope.

### Double-luciferase reporter assays

After BMSCs were transfected with lentiviruses containing the luciferase reporter gene, BMSCs were washed with PBS and lysed with cell lysate according to the instructions of the luciferase assay kit (Solarbio), and then, Renilla luciferase reaction substrate was added. Finally, luciferase activity was detected at a wavelength of 465 nm by a multifunction enzyme marker system (BioTek).

### RNA binding protein immunoprecipitation (RIP)

RIP was performed using a Magna RNA-binding protein immunoprecipitation kit (Merck Millipore, Billerica, CA, USA) according to the manufacturer’s instructions. First, Ago2 antibody-magnetic beads were incubated with cell lysates, and IgG negative controls were established at the same time. Then, coimmunoprecipitation was conducted to isolate all RNAs and proteins bound to Ago2. Finally, purified RNAs were extracted and determined by real-time PCR to confirm the presence of the binding targets.

### Real-time quantitative PCR

We extracted RNA from BMSCs via column affinity purification (QIAGEN, Hilden, Germany) and synthesized complementary DNAs (cDNAs) using M-MuLV RT Master Mix with Oligo(dT) (Sangon Biotech, Shanghai, China). We performed real-time PCR on a StepOnePlus system (Applied Biosystems, Foster City, CA, USA) in 96-well plates with specific primers and SYBR Green Mix (Sangon Biotech). The primers (Sangon Biotech) were as follows: Lnc Tmem235-F: GGGAGAAGGT CATCTCAGGCA; Lnc Tmem235-R: GCTGTTGCTGCCTTTCTCAAGT; Lnc LOC102553514-F: CAGCGTCAGACCTCCGTCTA; Lnc LOC102553514-R: TTAA GCATTGCGGGTGCCAA; BIRC5-F: TGCCTTACGCTGAGCCTTTGC; BIRC5-R: GCCTGGAAAGCTGGGACAAGTG; miRNA-34a-3p-F: CGCGCGAATCAGCAA GTATACT; miRNA-34a-3p-R: AGTGC AGGGTCCGAGGTATT; miRNA-34a-3p-RT: GTCGTATCCAGTGCAGGGTCCGAGGTATTCGCACTGGATACGACTAGGGC; ACTB-F: CACCCGCGAGTACAACCTTC; and ACTB-R: CCCATACCCACCATCA CACC. We calculated the fold change in RNA expression compared to that of the control using the ΔΔCt method.

### Western blotting

The cells were lysed with RIPA cell lysate (Beyotime) and centrifuged at 13000 g/min for 10 min. The supernatant was obtained, and the protein was quantified by a BCA protein concentration detection kit (Solarbio). SDS‒PAGE gels were prepared, the same amount of protein was added for electrophoresis, and then the protein was transferred to a PVDF membrane. The following antibodies were used for primary antibody reactions: rabbit anti-BIRC5 (1:3000; ab469; Abcam, Cambridge, MA, USA), rabbit anti-Bcl-2 (1:500; bs-0032R; Bioss, Beijing, China), rabbit anti-Caspase-3 (1:500; ab4051; Abcam), rabbit anti-Bax (1:1500; bs-28034R; Bioss), mouse anti-β-actin (1:2500; ab8226; Abcam), rabbit anti-CASP-3/Cleaved CASP-3 (1:500; ab13847; Abcam), rabbit anti-CASP-9/Cleaved CASP-9 (1:1000; ab25758; Abcam), rabbit anti-PARP1/Cleaved PARP1 (1:10000; ab191217; Abcam), rabbit anti-Argonaute 2 (1:800; ab5072; Abcam), mouse anti-OCN (10 µg/mL; ab13420; Abcam), rabbit anti-OPG (1:100; bs-20624R; Bioss), rabbit anti-Runx2 (1:200; AV36678; Sigma-Aldrich), and rabbit anti-OPN (1:200; ab8448; Abcam). HRP-conjugated mouse anti-rabbit IgG was used for secondary-antibody reactions. ECL (Merck Millipore) photoluminescence solution was used for exposure. Finally, the images were collected by a gel imaging system (Clinx Science Instruments, Ltd., Shanghai, China) and quantitatively analyzed by ImageJ software (1.4.3.67, NIH, Bethesda, MD, USA).

### Xenogeneic antigen-extracted cancellous bone (XACB)

We removed the bone cortex and cartilage from fresh porcine vertebrae, and the cancellous bone was made into a cylinder with a diameter of 5 mm and a height of 10 mm. The bone was washed repeatedly in an ultrasonic cleaning machine. Then, samples were deproteinized with 30% hydrogen peroxide (Chengdu Jinshan Chemical Reagent Co., Ltd., Chengdu, China) for 48 h while stirring intermittently and by replacing hydrogen peroxide every 24 h. The samples were then degreased with 1:1 chloroform/methanol (Chengdu Jinshan Chemical Reagent Co., Ltd.) for 24 h while stirring intermittently, and the 1:1 chloroform/methanol was replaced every 12 h. Samples were freeze-dried at −50 °C for 24 h and were sterilized with ethylene oxide for 24 h, followed by being aseptically packaged until further use.

### Early SONFH model

A total of 150 adult male SD rats were intravenously injected twice with lipopolysaccharides (2 mg/kg; Sigma-Aldrich) once a day. After the second injection of lipopolysaccharides, methylprednisolone (60 mg/kg; Pfizer, Andover, MA, USA) was injected seven times into the gluteal muscle immediately, once a day, and the rats were weighed before each injection.

### Biocompatibility of XACB with BMSCs

The XACB was made into a small cylinder with a diameter of 5 mm and a height of 5 mm, after which it was soaked in complete L-DMEM for 24 h. A BMSC suspension (1 × 10^7^ cells/mL) was dropped into XACB and incubated in an incubator at 37 °C and 5% CO_2_ for 3 h, and then, complete L-DMEM was slowly added along the wall of the culture plate to just pass XACB, after which the culture was continued in the incubator. On the sixth day, the growth of BMSCs on the surface of XACB was observed by scanning electron microscopy (Hitachi, Tokyo, Japan).

### Tissue-engineered bone

After Lv-Lnc Tmem235 and Lv-Sh-Lnc Tmem235 were transfected into BMSCs, BMSCs were dropped into XACB and cocultured according to the above steps to construct tissue-engineered bone.

### Tissue-engineered bone transplantation

The skin of the right hip of SD rats was cleaned one week before the operation. Penicillin (50000 U/kg; CSPC, Shijiazhuang, China) was injected intramuscularly 1 h before the operation to prevent infection. Then, 3% pentobarbital sodium (1 mL/kg; Merck Millipore) was injected intravenously to anesthetize the rats. The rats were then placed in a prone position, and the operated area was disinfected. A posterolateral straight incision of the hip joint was made to expose the hip joint, and then, the femoral head was rotated externally. At the junction of bone and cartilage, a sterile spherical drill with a diameter of 2 mm was used to grind this junction from the posterolateral to the anterior medial side, and the depth was approximately 3 mm. The necrotic bone tissue was completely scraped, and the tissue-engineered bone was implanted according to the experimental group, after which the pore was filled with a gelatin sponge (Alicon, Hangzhou, China). There were 18 rats in each group. Penicillin was used for 3 d after the operation to prevent infection.

### Live imaging of animals

The BMSCs were labeled with DiR (Yeasen, Shanghai, China) fluorescent dyes before the operation. At 48 h after BMSC transplantation, the fluorescence intensity of the femoral head necrosis area was detected by a small animal imaging system (PerkinElmer, MA, USA). Chloral hydrate (10%, 3 mL/kg) was administered in the abdominal cavity, and the rats were placed on an imaging platform. Fluorescence images were obtained, and the fluorescence intensity was calculated.

### Micro-computed tomography (micro-CT)

Twelve weeks after BMSC transplantation, the femoral head tissue was scanned using the Bruker micro-CT system (SkyScan1276, Bruker, Karlsruhe, Germany) under uniform conditions (voltage 85 kV, current 200 μA, resolution 6.5 μm). N-Recon software (V1.7.4.2, Bruker) was used for 3D image reconstruction, and CT Analyser software (1.18.8.0, Bruker) was used for 3D analysis. Moreover, the trabecular number, trabecular thickness, new bone volume, and volume fraction were detected.

### H&E and Masson staining

The femoral head was fixed with 4% formaldehyde (Solarbio), decalcified with 10% EDTA (Solarbio), dehydrated with gradient alcohol (SINOPHARM, Beijing, China), made transparent using xylene (SINOPHARM), embedded in paraffin (SINOPHARM), and sliced with a Leica pathological slicer (RM2016, Leica, Wetzlar, Germany). The slices were dewaxed in xylene and gradient alcohol in turn. The dewaxed sections were stained according to the HE staining kit (Solarbio) and the Masson staining kit (Solarbio) and then examined under a biomicroscopy (Olympus, Tokyo, Japan).

### TUNEL staining

The bone tissue was fixed, decalcified, dehydrated, made transparent, embedded, sliced, and dewaxed (the procedure was the same as that outlined for H&E staining). Then, the sections were stained according to the instructions of the TUNEL detection kit (Vazyme, Nanjing, China) and sealed with an anti-fluorescence quencher (Solarbio). The images were collected by a biomicroscopy (Olympus).

### Immunofluorescence

The bone tissue was fixed, decalcified, dehydrated, made transparent, embedded, sliced, and dewaxed (the procedure was the same as that outlined for H&E staining). Antigen retrieval was performed using the bone tissue antigen retrieval solution (BioShun, Shanghai, China). Then, bone tissues were blocked with goat serum (Solarbio). Rabbit anti-BIRC5 (1/100; ab134170; Abcam) and rabbit anti-GFP (1/1000; ab6556; Abcam) were used for primary antibody reactions. Goat anti-rabbit IgG (Alexa Fluor® 488, 1/800, ab150077, Abcam) and goat anti-rabbit IgG (Alexa Fluor® 594, 1/800, ab150080, Abcam) were used for secondary antibody reactions. DAPI restaining of the nucleus was performed, and the sections were sealed with a sealing solution containing an anti-fluorescent quenching agent (Solarbio), after which images were observed and collected under a biomicroscopy (Olympus).

### Statistical analysis

SPSS 22.0 statistical software was used to analyze the data, and GraphPad Prism 6.0 software was used to draw statistical graphs. For quantitative data, we used a Kolmogorov–Smirnov test to analyze the normality of the data and ANOVA to test the homogeneity of variance of the data between groups. The data were normally distributed, and the variance was homogeneous. Data are expressed as the means ± standard deviations (SDs). Two-tailed unpaired Student’s t tests were used for analyses involving only two groups for comparison, and one-way ANOVA with Tukey’s *post-hoc* test was used for analyses involving more than two groups. The data were not normally distributed and are expressed as M (P25, P75). The Kruskal–Wallis rank-sum test was used for comparisons between groups. If the difference between groups was statistically significant, the DSCF method was further used for pairwise comparisons between groups. *P* < 0.05 was considered statistically significant.

## Results

### The expression of Lnc Tmem235 is downregulated during hypoxia-induced apoptosis of BMSCs

**Fig. 1:**
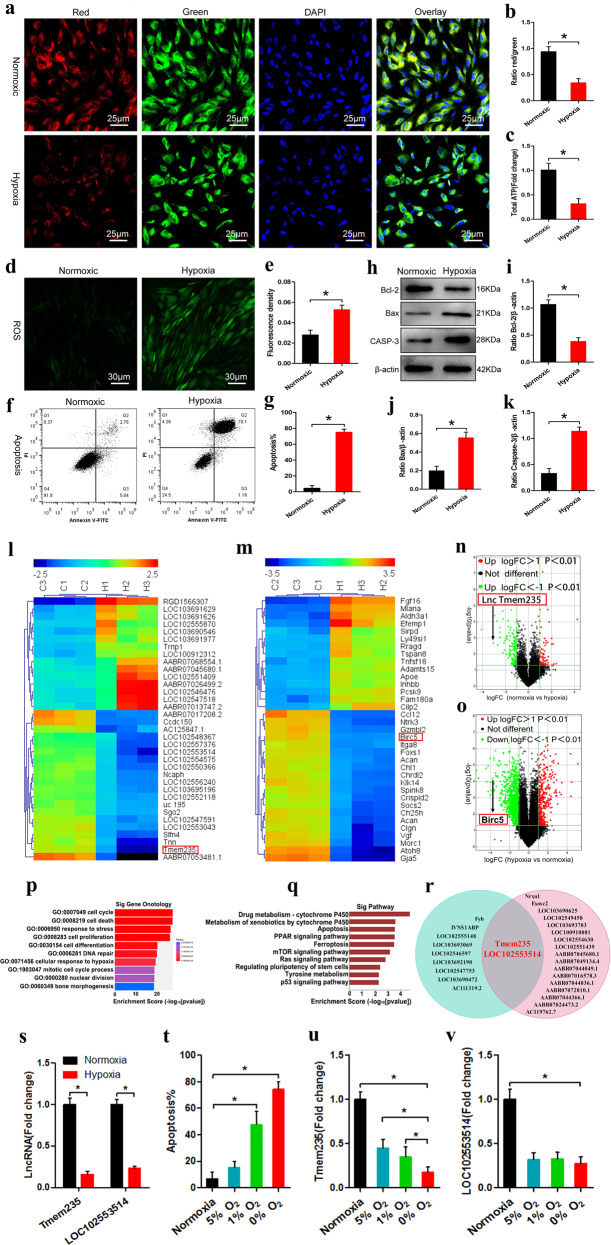
The expression of Lnc Tmem235 is downregulated during hypoxia-induced apoptosis in BMSCs. **a**, **b** JC-1 was used to detect the mitochondrial membrane potential (*n* = 5); bone-marrow mesenchymal stem cells (BMSCs), 5,5′,6,6′ - tetrachloro − 1,1′,3,3′ - tetraethyl - imidacarbocyanine iodide (JC-1), and 4′,6 - diamidino - 2 - phenylindole (DAPI); **c** ATP content (*n* = 6); adenosine triphosphate (ATP); **d**, **e** The content of ROS was detected by DCFH-DA (n = 5); reactive oxygen species (ROS) and 2′,7′ - dichlorofluorescin diacetate (DCFH-DA); **f**, **g** Apoptosis was detected by Annexin V/PI (*n* = 5); fluorescein isothiocyanate (FITC) and propidium iodide (PI); **h**–**k** The expression levels of Bcl-2, Bax, and CASP3 were analyzed by western blotting (*n* = 3); B-cell lymphoma 2 (Bcl-2), Bcl-2-associated X protein (Bax), and Caspase-3 (CASP-3); **l** Cluster analysis of lncRNAs (*n* = 3); **m** Cluster analysis of mRNAs (*n* = 3); **n** Volcano map of lncRNA expression profile (*n* = 3); fold change (FC), the base of logFC is 2; **o**. Volcano map of mRNA expression profile (*n* = 3); the base of logFC is 2; **p** GO analysis (*n* = 3); Gene Ontology (GO); **q** KEGG analysis (*n* = 3); Kyoto Encyclopedia of Genes and Genomes (KEGG); **r** Coexpression analysis and gene-position relationships were used to screen candidate lncRNAs; **s** The expression levels of Lnc Tmem235 and Lnc LOC102553514 were verified by qPCR (*n* = 6); real-time quantitative polymerase chain reaction (qPCR); **t** Apoptotic changes occurred as a function of hypoxia (*n* = 5); **u** Lnc Tmem235 changed with the degree of hypoxia (*n* = 6); **v** Lnc LOC102553514 changed with the degree of hypoxia. In (**b**, **c**, **e**, **g**, **i**–**k**, **s**–**v**), the data are normally distributed, and the variance is homogeneous. Data are presented as the means ± standard deviations (SDs). In (**b**, **c**, **e**, **g**, **i**–**k**, **s**), statistical significance was calculated by Student’s *t* tests; in (**t**–**v**), statistical significance was calculated by one-way ANOVA with Tukey’s post-*hoc* tests; ^*^P < 0.05.

We successfully isolated and cultured BMSCs from bone marrow (Supplementary Fig. 1) and explored the effects of different hypoxic environments on BMSCs. We set the oxygen concentration gradients to 20%, 10%, 5%, 1%, and 0% and subjected the cells to hypoxia for 48 h. By detecting the mitochondrial membrane potential (Supplementary Fig. 2a, b), ATP content (Supplementary Fig. 2c), ROS level (Supplementary Fig. 2d, e), osteogenic activity (Supplementary Fig. 2f, g), cell apoptosis (Supplementary Fig. 2h–l), and cell viability (Supplementary Fig. 2m), we demonstrated that an appropriate hypoxic environment had no significant effect on BMSCs and may even improve cell viability and osteogenic activity. However, when the oxygen concentration was <1%, the cells suffered irreversible damage and apoptosis (Supplementary Fig. 2). Because the oxygen concentration in the necrotic area of the femoral head was < 1%, we used an oxygen concentration of 0% to establish the hypoxia model of BMSCs in vitro. We subjected BMSCs to hypoxia (0% O_2_, 95% N_2_, and 5% CO_2_) for 48 h. The results showed that after BMSCs were exposed to hypoxia, the mitochondrial membrane potential and ATP levels were decreased (Fig. 1a–c), while ROS levels were increased (Fig. 1d, e); furthermore, the expression of B-cell lymphoma-2 (Bcl-2) was downregulated, Caspase-3 (CASP-3) and Bcl-2-related X (Bax) were upregulated, and a large number of BMSCs were apoptotic (Fig. 1f–k).

LncRNAs can regulate gene expression in many different ways and play important roles in the regulation of apoptosis^22–25^. Hence, we next determined which lncRNAs were related to hypoxia-induced apoptosis of BMSCs. First, we cultured BMSCs under hypoxia (0% O_2_, 95% N_2_ and 5% CO_2_) for 48 h and then searched for hypoxia-reactive lncRNAs and mRNAs via a lncRNA chip. The results showed that 99 lncRNAs were upregulated, 183 lncRNAs were downregulated (Fig. 1l, n), 422 mRNAs were upregulated, and 1104 mRNAs were downregulated (fold change > 2, *P* < 0.05) (Fig. 1m, o). Then, Gene Ontology (GO) and Kyoto Encyclopedia of Genes and Genomes (KEGG) pathway analyses were performed on these differentially expressed mRNAs to determine apoptosis-related mRNAs (fold change > 4, *P* < 0.01) (Fig. 1p–q). We then analyzed mRNA/lncRNA coexpression (abs ≥ 0.98, *P* value ≤ 0.01, FDR ≤ 1%) and corresponding gene-location relationships (distance < 200 kb). We identified two apoptosis-related candidate lncRNAs: Tmem235 and LOC102553514 (Fig. 1r). Next, we used quantitative real-time PCR (qPCR) to verify the expression of candidate lncRNAs in our BMSC hypoxia model, and we found that the results were consistent with our microarray data (Fig. 1s). Among them, Lnc Tmem235 expression was significantly downregulated, and its expression was continuously downregulated with the aggravation of hypoxia and the concomitantly increased apoptotic rate, while the expression of Lnc LOC102553514 was not related to the degree of hypoxia (Fig. 1t–v). These results suggest that Lnc Tmem235 may be related to hypoxia-induced apoptosis of BMSCs.

### Lnc Tmem235 inhibits hypoxia-induced apoptosis of BMSCs

**Fig. 2:**
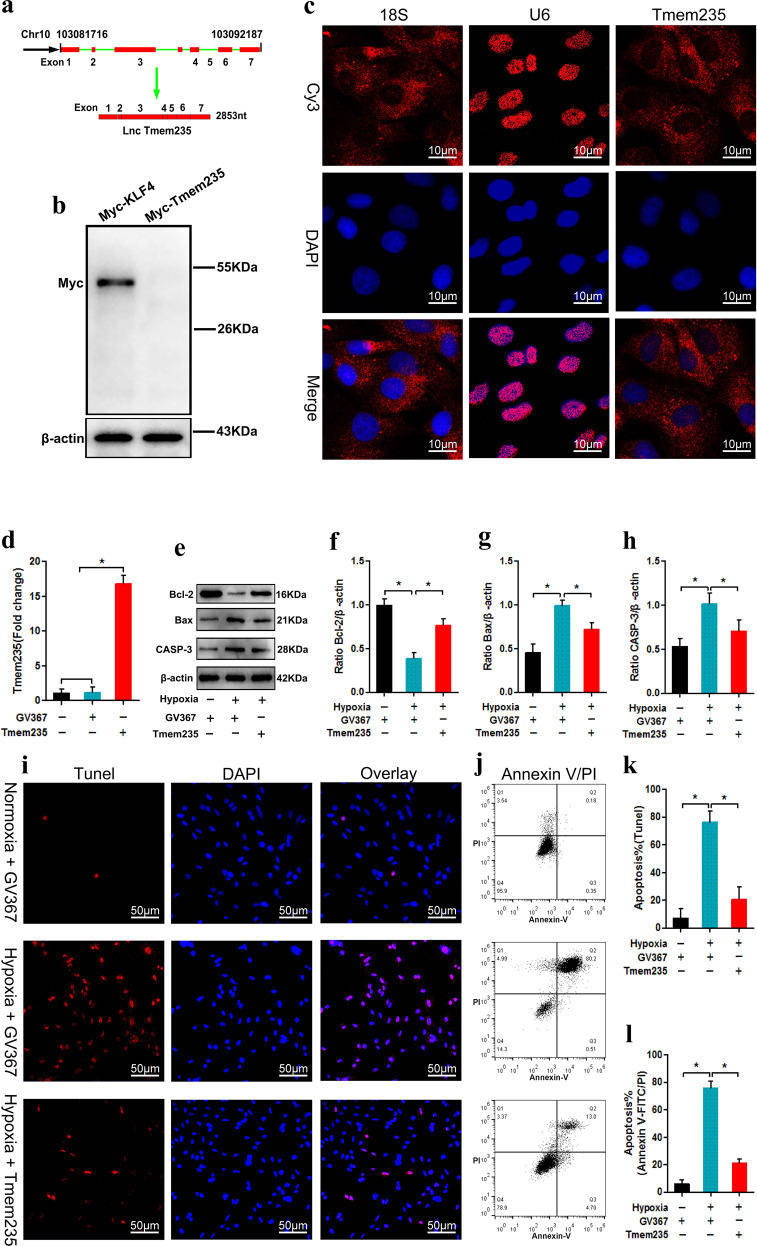
Lnc Tmem235 inhibits hypoxia-induced apoptosis of BMSCs. **a** Localization of Lnc Tmem235 in the genome; **b** Western blot detection of myc fusion protein to evaluate the coding ability of Lnc Tmem235 (*n* = 3), and KLF4 was used as the coding protein control; Kruppel-like factor-4 (KLF4); **c** RNA-FISH detection of subcellular localization of Lnc Tmem235 (*n* = 5), and 18 S and U6 were used as positive controls for the cytoplasm and nucleus, respectively; RNA fluorescence in situ hybridization (RNA-FISH); **d** The expression of Lnc Tmem235 was detected by qPCR (*n* = 6); empty vector (GV367); **e**–**h** The expression levels of Bcl-2, Bax, and CASP3 were analyzed by western blotting (*n* = 3); **i**, **k** Apoptosis was detected by TUNEL/DAPI (*n* = 5); TdT-mediated dUTP nick-end labeling (TUNEL); **j**, **l** Apoptosis was detected by Annexin V/PI (*n* = 5). In (**d**, **f**–**h**, **k**–**l**), the data are normally distributed, and the variance is homogeneous. Data are presented as the means ± SDs; statistical significance was calculated by one-way ANOVA with Tukey’s post-*hoc* tests; ^*^P < 0.05.

The Lnc Tmem235 locus is located on chromosome 10, downstream of BIRC5, and has the same transcriptional direction as BIRC5. This locus consists of seven exons, and the full length of the transcript is 2853 nt (Fig. 2a). We cloned the Lnc Tmem235 transcript into the pcDNA4/myc-hisplasmid and analyzed the expression of myc fusion protein by immunoblotting with an anti-myc antibody. Kruppel-like factor-4 (Klf4) was used as the protein control. The results showed that Lnc Tmem235 did not have the ability to encode the protein (Fig. 2b). We then used RNA-FISH to determine the subcellular location of Lnc Tmem235; 18 S and U6 were used as positive controls for the cytoplasm and nucleus, respectively. The results showed that Lnc Tmem235 was mainly distributed in the cytoplasm of BMSCs (Fig. 2c).

To further study the effects of Lnc Tmem235 on hypoxia-induced apoptosis of BMSCs, we transfected BMSCs with an Lnc Tmem235 overexpression lentivirus (Lv-Lnc Tmem235) to overexpress Lnc Tmem235 (Fig. 2d). Then, BMSCs were treated with hypoxia (0% O_2_, 95% N_2_, and 5% CO_2_) for 48 h. The results showed that under hypoxia, the expression levels of Lnc Tmem235 and Bcl-2 were downregulated, while the expression levels of Bax and CASP-3 were upregulated (Fig. 2e–h), and the BMSC apoptotic rate was more than 70% (Fig. 2i–l). However, overexpression of Lnc Tmem235 reversed the above results, as it significantly reduced the apoptotic rate of BMSCs and promoted the survival of BMSCs under hypoxia (Fig. 2e–l). These findings suggest that Lnc Tmem235 inhibited hypoxia-induced apoptosis of BMSCs. However, the mechanism by which Lnc Tmem235 inhibits hypoxia-induced apoptosis in BMSCs remains unclear.

### Lnc Tmem235 inhibits hypoxia-induced apoptosis of BMSCs by regulating BIRC5

**Fig. 3:**
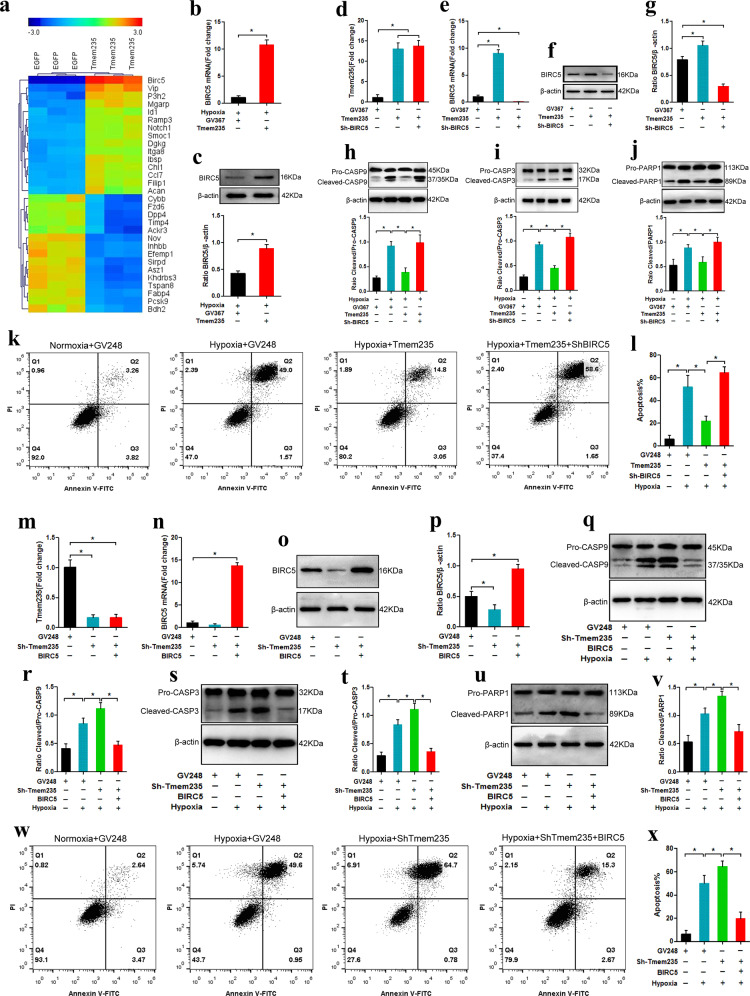
Lnc Tmem235 inhibits hypoxia-induced apoptosis of BMSCs by regulating BIRC5. **a** mRNA clustering analysis (*n* = 3); **b** The expression of BIRC5 mRNA was detected by qPCR (*n* = 3); baculoviral inhibitor of apoptosis (IAP) repeat-containing 5 (BIRC5); **c** Western blotting was used to detect the expression of BIRC5 protein (*n* = 3); **d** The expression of Lnc Tmem235 was detected by qPCR (*n* = 3); **e** The expression of BIRC5 mRNA was detected by qPCR (*n* = 3); **f**, **g** Western blotting was used to detect the expression of BIRC5 protein (*n* = 3); **h** The activity of CASP-9 was analyzed by western blotting (*n* = 3); Caspase-9 (CASP-9); **i**. The activity of CASP-3 was analyzed by western blotting (*n* = 3); **j** The fragmentation of PARP1 was analyzed by western blotting (*n* = 3); poly (ADP-ribose) polymerase 1 (PARP1); **k**, **l** Apoptosis was detected by Annexin V/PI (*n* = 5); empty vector (GV248); **m**. The expression of Lnc Tmem235 was detected by qPCR (*n* = 3); **n** The expression of BIRC5 mRNA was detected by qPCR (*n* = 3); **o**, **p** The expression of BIRC5 protein was detected by western blotting (*n* = 3); **q**, **r** The activity of CASP-9 was analyzed by western blotting (*n* = 3); **s**–**t** The activity of CASP-3 was analyzed by western blotting (*n* = 3); **u**, **v** The fragmentation of PARP1 was analyzed by western blotting (*n* = 3); **w**, **x**. Apoptosis was detected by Annexin V/PI (*n* = 5). In (**b**–**e**, **g**–**j**, **l**–**n**, **p**, **r**, **t**, **v**, **x**), the data are normally distributed, and the variance is homogeneous. Data are presented as the means ± SDs; in (**b**–**c**), statistical significance was calculated with Student’s *t* tests; in (**d**, **e**, **g**–**j**, **l**–**n**, **p**, **r**, **t**, **v**, **x**), statistical significance was calculated by one-way ANOVA with Tukey’s post-*hoc* tests; ^*^P < 0.05.

To elucidate the mechanism by which Lnc Tmem235 inhibited hypoxia-induced apoptosis of BMSCs, we overexpressed Lnc Tmem235 in BMSCs (BMSCs-Lnc Tmem235) and subjected them to hypoxia (0% O_2_, 95% N_2_ and, 5% CO_2_) for 48 h. Then, we detected the gene expression profile of BMSCs-Lnc Tmem235 by microarray analysis to screen the downstream genes that may be regulated by Lnc Tmem235. The results showed that 156 mRNAs were upregulated and 659 mRNAs were downregulated (fold change > 2, *P* < 0.05), among which the expression of bacterial inhibitor of apoptosis (IAP) repeat-containing 5 (BIRC5) was significantly upregulated (Fig. 3a). Then, we used qPCR to verify the microarray data, and the results showed that Lnc Tmem235 promoted the expression of BIRC5 in BMSCs under hypoxia (Fig. 3b, c). BIRC5, as an apoptotic inhibitor, can directly inhibit the activities of CASP-3 and CASP-9, thereby blocking apoptosis^33–36^. Therefore, we speculated that Lnc Tmem235 may have inhibited hypoxia-induced apoptosis of BMSCs by regulating the expression of BIRC5.

We next determined whether Lnc Tmem235 inhibited hypoxia-induced apoptosis of BMSCs via regulation of BIRC5. We first transfected BMSCs with Lv-Lnc Tmem235 to upregulate Lnc Tmem235 (Fig. 3d). Then, we subjected BMSCs to hypoxia (0% O_2_, 95% N_2_, and 5% CO_2_) for 48 h. We found that the activities of CASP-3 and CASP-9 and hypoxia-induced apoptosis of BMSCs were decreased significantly after upregulation of Lnc Tmem235 (Fig. 3h–l). Then, on the basis of upregulating Lnc Tmem235, we further transfected BMSCs with a BIRC5 interference lentivirus, which resulted in the downregulation of BIRC5 expression (Fig. 3e–g). The results showed that the downregulation of BIRC5 significantly weakened the antiapoptotic effect of Lnc Tmem235 (Fig. 3h–l). In contrast, we transfected BMSCs with Lv-Sh-Lnc Tmem235, which significantly decreased the expression of Lnc Tmem235 (Fig. 3m), and the activities of CASP-3 and CASP-9 and hypoxia-induced apoptosis of BMSCs were increased significantly after Lnc Tmem235 was downregulated (Fig. 3q–x). Then, on the basis of downregulating Lnc Tmem235, we further transfected BMSCs with a BIRC5 overexpression lentivirus (Lv-BIRC5), which resulted in upregulation of BIRC5 expression (Fig. 3n–p). The results showed that upregulation of BIRC5 effectively ameliorated hypoxia-induced apoptosis of BMSCs (Fig. 3q–x). These results confirmed that Lnc Tmem235 inhibited hypoxia-induced apoptosis of BMSCs by regulating BIRC5 expression. However, it remains unclear how LncTmem235 regulates the expression of BIRC5.

### Lnc Tmem235 regulates BIRC5 expression by targeting miR-34a-3p

**Fig. 4:**
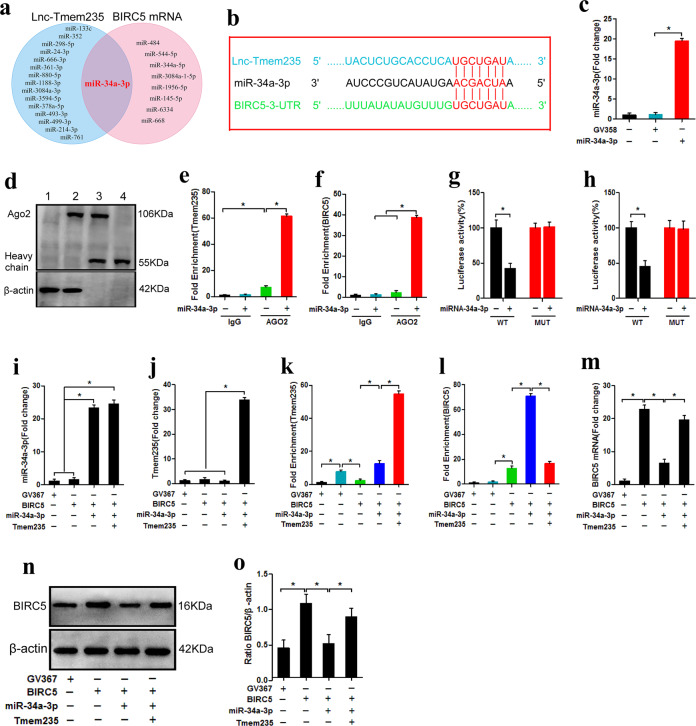
Lnc Tmem235 regulates BIRC5 expression by targeting miR-34a-3p. **a** MiRDB and RNAhybrid predicted the miRNA of the possible combination of Lnc Tmem235 and BIRC5 mRNA 3′UTR; untranslated region (UTR) and microRNA (miRNA); **b** Binding sites of miR-34a-3p with Lnc Tmem235 and BIRC5 mRNA 3′UTR; **c**. The expression of miR-34a-3p was detected by qPCR (*n* = 4); empty vector (GV358); **d**–**f** RIP detection of enrichment of Lnc Tmem235 and BIRC5 mRNA to miRNPs (*n* = 3); RNA-binding protein immunoprecipitation (RIP); **g**, **h** Luciferase activity analysis of the binding of miR-34a-3p to Lnc Tmem235 or BIRC5 mRNA (*n* = 5); **i** The expression of miR-34a-3p was detected by qPCR (*n* = 3); **j**. The expression of Lnc Tmem235 was detected by qPCR (*n* = 3); **k** RIP detection of enrichment of Lnc Tmem235 to miRNPs (*n* = 3); miRNA ribonucleoprotein complexes (miRNPs); **l** RIP detection of enrichment of BIRC5 mRNA to miRNPs (*n* = 3); **m** The expression of BIRC5 mRNA was detected by qPCR (n = 3); **n**, **o** Western blotting was used to detect the expression of BIRC5 protein (*n* = 3). In (**c**, **e**–**m**, **o**), the data are normally distributed, and the variance is homogeneous. Data are presented as the means ± SDs; statistical significance was calculated by one-way ANOVA with Tukey’s post hoc tests; ^*^P < 0.05.

Studies have shown that lncRNAs can competitively bind to miRNAs with target gene mRNAs, thus releasing the silencing effect of miRNAs on target genes to ultimately promote the expression of target genes^37^. Hence, we next investigated whether Lnc Tmem235 regulated the expression of BIRC5 through the above mechanism. We used bioinformatic tools (miRDB and RNAhybrid) to predict possible binding miRNAs of Lnc Tmem235 and untranslated regions at the 3’ end of BIRC5 mRNA (BIRC5 mRNA 3’UTR). The results showed that miR-34a-3p could simultaneously bind to Lnc Tmem235 and the BIRC5 mRNA 3’UTR (Fig. 4a) and that it had the same binding site on miR-34a-3p. Thus, Lnc Tmem235 and BIRC5 mRNA satisfied the conditions for competitive binding to miR-34a-3p (Fig. 4b). Hence, we speculated that Lnc Tmem235 may competitively bind to miR-34a-3p with BIRC5 mRNA, thus releasing the silencing effect of miR-34a-3p on BIRC5 mRNA to ultimately promote the expression of BIRC5.

We next determined whether miR-34a-3p could simultaneously combine with Lnc Tmem235 and BIRC5 mRNA. First, we overexpressed miR-34a-3p in BMSCs (Fig. 4c) and detected the enrichment of Lnc Tmem235 and BIRC5 mRNA in miRNA ribonucleoprotein complexes (miRNPs) via RIP. The results showed that overexpression of miR-34a-3p significantly increased the enrichment of Lnc Tmem235 and BIRC5 mRNA in miRNPs (Fig. 4d–f). Then, we inserted the cDNA of Lnc Tmem235 and the BIRC5 mRNA 3’UTR downstream of the luciferase (Luc) reporter gene to construct overexpression lentiviruses (Lv-Luc-Lnc Tmem235 and Lv-Luc-BIRC5 3’UTR) to transfect BMSCs. Moreover, BMSCs were transfected with a miR-34a-3p overexpression lentivirus (Lv-miR-34a-3p). Our luciferase-reporter assay showed that luciferase activity was decreased significantly after miR-34a-3p was upregulated (Fig. 4g, h). When we mutated the predicted binding sites of Lnc Tmem235 or BIRC5 mRNA 3’UTR on miR-34a-3p (Lnc Tmem235 MUT and BIRC5 3’UTR MUT), we used the same method to construct overexpression lentiviruses (Lv-Luc-Lnc Tmem235 MUT and Lv-Luc-BIRC5 3’UTR MUT) to transfect BMSCs. At this time, our luciferase reporter assay showed that upregulation of miR-34a-3p expression had no significant effect on luciferase activity (Fig. 4g, h). These results indicate that miR-34a-3p can bind to the predicted sites on Lnc Tmem235 or BIRC5 mRNA.

We next investigated whether Lnc Tmem235 can competitively bind to miR-34a-3p with BIRC5 mRNA to regulate the expression of BIRC5. We first transfected BMSCs with Lv-BIRC5 to overexpress BIRC5 and then transfected BMSCs with Lv-miR-34a-3p to upregulate the expression of miR-34a-3p (Fig. 4i). Finally, the enrichment of BIRC5 mRNA in miRNPs was detected by RIP, and the mRNA and protein levels of BIRC5 were detected by qPCR and western blotting, respectively. The results showed that BIRC5 mRNA was significantly enriched in miRNPs, whereas the expression of BIRC5 was downregulated (Fig. 4l–o). Furthermore, on the basis of upregulating miR-34a-3p, we further upregulated Lnc Tmem235 (Fig. 4j); the results showed that Lnc Tmem235 was significantly enriched in miRNPs, the enrichment of BIRC5 mRNA in miRNPs decreased, and BIRC5 expression was upregulated (Fig. 4k–o).

The above results confirmed that Lnc Tmem235 could be used as a ceRNA to competitively bind to miR-34a-3p with BIRC5 mRNA, thereby relieving the silencing effect of miR-34a-3p on BIRC5 mRNA and ultimately promoting the expression of BIRC5.

### Lnc Tmem235 inhibits hypoxia-induced apoptosis of BMSCs by regulating miR-34a-3p/BIRC5

**Fig. 5:**
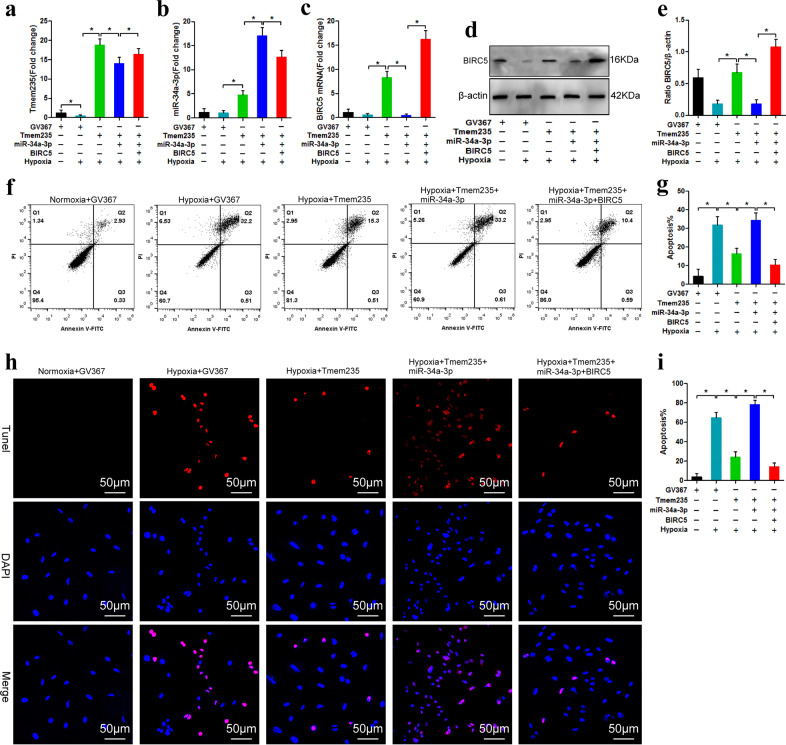
Lnc Tmem235 inhibits hypoxia-induced apoptosis of BMSCs by regulating miR-34a-3p/BIRC5. **a** The expression of Lnc Tmem235 was detected by qPCR (*n* = 4). **b** The expression of miR-34a-3p was detected by qPCR (*n* = 4). **c** The expression of BIRC5 mRNA was detected by qPCR (*n* = 4). **d**, **e** Western blotting was used to detect the expression of BIRC5 protein (*n* = 3). **f**, **g** Apoptosis was detected by Annexin V/PI (*n* = 5). **h**, **i** Apoptosis was detected by TUNEL/DAPI (*n* = 5). In (**a**–**c**, **e**, **g**, **i**), the data are normally distributed, and the variance is homogeneous. Data are presented as the means ± SDs; statistical significance was calculated by one-way ANOVA with Tukey’s *post*-*hoc* tests; ^*^P < 0.05.

We next determined whether Lnc Tmem235 inhibited hypoxia-induced apoptosis of BMSCs by regulating miR-34a-3p/BIRC5. We transfected BMSCs with Lv-Lnc Tmem235 to overexpress Lnc Tmem235 in BMSCs (Fig. 5a) and then subjected BMSCs to hypoxia (0% O_2_, 95% N_2_, and 5% CO_2_) for 48 h. Compared with that of the Lv-EGFP group, the expression of BIRC5 in the Lv-Lnc Tmem235 group was upregulated (Fig. 5c–e), and apoptosis was significantly reduced (Fig. 5f–i). When we upregulated the expression of miR-34a-3p on the basis of overexpression of Lnc Tmem235 (Fig. 5b), the content of BIRC5 was decreased, and apoptosis was increased. The results showed that miR-34a-3p blocked the antiapoptotic effect of Lnc Tmem235 (Fig. 5c–i). Subsequently, on the basis of overexpression of Lnc Tmem235 and miR-34a-3p, we upregulated the expression of BIRC5. Following this manipulation, apoptosis was reduced, revealing that upregulation of BIRC5 rescued BMSCs from hypoxia-induced apoptosis (Fig. 5c–i). These results confirmed that Lnc Tmem235 inhibited hypoxia-induced apoptosis of BMSCs by regulating the miR-34a-3p/BIRC5 axis.

### Lnc Tmem235 inhibits hypoxia-induced apoptosis of BMSCs and promotes repair of early SONFH

**Fig. 6:**
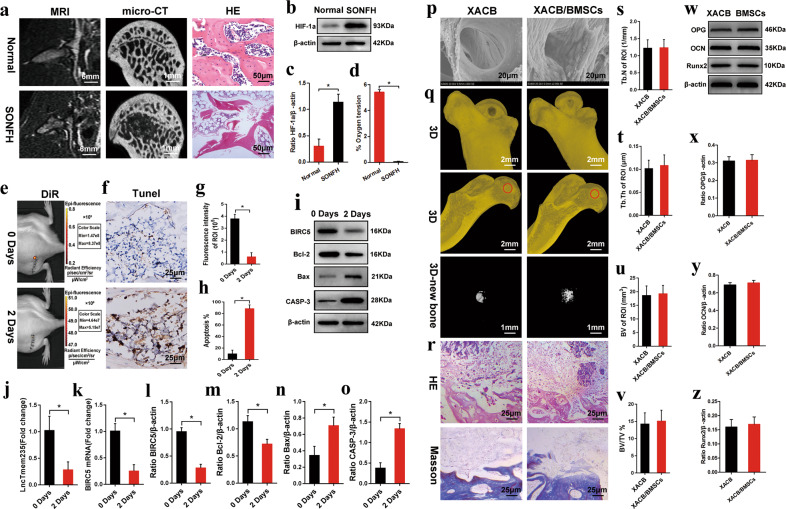
Transplantation of BMSCs to repair early SONFH. **a** MRI, micro-CT, and HE staining were used to evaluate the SONFH model; magnetic resonance imaging (MRI), microcomputed tomography (micro-CT), and steroid-induced osteonecrosis of the femoral head (SONFH); **b**–**c** The expression of HIF-1α in the femoral head necrotic area was detected by western blotting (*n* = 6); hypoxia inducible factor 1 alpha (HIF-1α); **d** Direct detection of oxygen concentration in the femoral head necrotic area (*n* = 6); **e** At 0 and 2 days after surgery, the DiR fluorescence intensity in the transplanted area was detected by live imaging of small animals (*n* = 8); 1,1-dioctadecyl-3,3,3,3-tetramethylindotricarbocyanine iodide (DiR); **f** At 0 and 2 days after surgery, apoptosis of transplanted cells was detected by TUNEL staining (*n* = 6); **g** Quantitative analysis of the DiR fluorescence intensity in the transplanted area as shown in **e** (*n* = 8); **h** Quantitative analysis of the proportion of TUNEL positive cells in the transplanted area as shown in **f** (*n* = 6); **i** At 0 and 2 days after surgery, the expression levels of BIRC5, Bcl-2, Bax, and CASP3 in the femoral head necrotic area were detected by western blotting (*n* = 6); **j** The expression levels of Lnc Tmem235 in the femoral head necrotic area were detected by qPCR (*n* = 6); **k** The expression levels of BIRC5 mRNA in the femoral head necrotic area were detected by qPCR (*n* = 6); **l** Quantitative analysis of BIRC5 expression as shown in **i** (*n* = 6); **m** Quantitative analysis of Bcl-2 expression as shown in **i** (*n* = 6); **n** Quantitative analysis of Bax expression as shown in **i** (*n* = 6); **o** Quantitative analysis of CASP-3 expression as shown in **i** (*n* = 6); **p** Scanning electron microscope observation of tissue-engineered bone XACB/BMSCs (*n* = 8); xenogeneic antigen-extracted cancellous (XACB) and tissue-engineered bone (XACB/BMSCs). **q** At 12 weeks after surgery, micro-CT analysis of the repair of the necrotic area of the femoral head (*n* = 6); red circle indicates the transplantation area; **r** At 12 weeks postsurgery, H&E and Masson staining were used to evaluate the repair of the necrotic area (*n* = 6); hematoxylin-eosin (H&E); **s** Quantitative analysis of the number of trabeculae as shown in **q** (*n* = 6); **t** Quantitative analysis of the trabecular thickness as shown in **q** (*n* = 6); **u** Quantitative analysis of the volume of new bone tissue as shown in **q** (*n* = 6); **v** Quantitative analysis of the volume fraction of new bone tissue as shown in **q** (*n* = 6); **w** At 12 weeks after surgery, the expression levels of OPG, OCN, and Runx2 in the femoral head necrotic area were detected by western blotting (*n* = 6); osteoprotegerin (OPG), osteocalcin (OCN), and runt-related transcription factor 2 (Runx2); **x** Quantitative analysis of OPG expression as shown in **w** (*n* = 6); **y** Quantitative analysis of OCN expression as shown in **w** (*n* = 6); **z** Quantitative analysis of Runx2 expression as shown in **w** (*n* = 6). In (**c**, **d**, **g**, **h**, **j**–**o**, **s**–**v**, **x**–**z**), the data are normally distributed, and the variance is homogeneous. Data are presented as the means ± SDs; statistical significance was calculated by Student’s t test; ^*^P < 0.05.

We used lipopolysaccharides (10 μg/kg/d) combined with methylprednisolone (40 mg/kg/d) to establish an early SONFH model. At the sixth week of modeling, magnetic resonance imaging (MRI; T2-weighted image [WI]) showed mixed signals of different heights in the femoral head; micro-CT indicated obvious osteonecrosis in the femoral head, and hematoxylin and eosin (H&E) staining revealed that the medullary cavity was filled with a large amount of adipose tissue, the trabecular bone had become thinner, and no osteoblast-related cells were present (Fig. 6a). These results confirmed the success of our early SONFH model. The expression of HIF-1α was significantly upregulated in the necrotic area of the femoral head, and the oxygen concentration was lower than 0.1%. A hypoxic microenvironment was formed in the osteonecrotic area (Fig. 6b–d). To evaluate the therapeutic effect of BMSCs on early SONFH, we used 1,1-dioctadecyl-3,3,3,3-tetramethylindotricarbocyanine iodide (DiR) to label BMSCs, cocultured BMSCs with XACB to construct tissue-engineered bone (XACB/BMSCs) (Fig. 6p), and transplanted BMSCs to repair early SONFH. At 2 days post-surgery, the fluorescence intensity of DiR was significantly decreased (Fig. 6e, g), the expression of Lnc Tmem235, BIRC5, and Bcl-2 was downregulated, the expression of Bax and CASP3 was upregulated (Fig. 6i–o), and the proportion of TUNEL-positive cells was more than 80% in the BMSC transplantation group (Fig. 6f, h), which confirmed that a large number of BMSCs were apoptotic in the hypoxic environment of the osteonecrotic area. At 12 weeks post-surgery, compared with that of the XACB group, there was no significant repair of the osteonecrotic area or formation of new bone tissue in the XACB/BMSC group (Fig. 6q–r), and there were no significant differences in the number of trabeculae (Tb.N), trabecular thickness (Tb.Th), new bone volume (BV), new bone volume fraction (BVF) (Fig. 6s–v), or osteogenic markers, such as OPG, OCN, and Runx2 (Fig. 6w–z). These results indicate that a large number of transplanted BMSCs have hypoxia-induced apoptosis in the hypoxic microenvironment of the osteonecrotic area, which seriously limits the effect of BMSC transplantation.

Subsequently, we evaluated the repair effect of BMSCs on SONFH under hypoxic and nonhypoxic conditions. We transplanted BMSCs into the osteonecrotic area of SONFH (hypoxic environment) and the bone defect area of the normal femoral head (physiological oxygen concentration), with SONFH as the negative control and the normal femoral head as the positive control. Forty-eight hours after surgery, the oxygen concentration in the transplanted area and the apoptosis of transplanted BMSCs were detected. The results showed that the oxygen concentration in the bone defect area of the normal femoral head was >5% (Supplementary Fig. 3c), the expression level of HIF-1α was extremely low (Supplementary Fig. 3a, b), and the local area had a physiological oxygen concentration. The oxygen concentration in the osteonecrotic area of SONFH was significantly lower than 1% (Supplementary Fig. 3c), the expression level of HIF-1α was significantly increased (Supplementary Fig. 3a, b), and the local area was hypoxic. Compared with that in the normal/BMSC group, the apoptosis of the transplanted BMSCs in the SONFH/BMSC group was significantly increased, and the apoptotic rate was >80% (Supplementary Fig. 3d, e). Twelve weeks after surgery, the repair effect of bone necrosis and bone defects was evaluated. Compared with the SONFH group, the osteonecrotic area was not significantly repaired in the SONFH/BMSC group, and there were no significant differences in BVF, Tb.N, and Tb.Th (Supplementary Fig. 3f–i), and HE and Masson’s staining showed no obvious new bone formation (Supplementary Fig. 3j, k). Compared with those in the SONFH/BMSC group, the bone defects in the normal/BMSC group were completely repaired, and the BVF, Tb.N, and Tb.Th were significantly increased (Supplementary Fig. 3f–i), and HE and Masson’s staining showed that the bone defects were completely filled with new bone tissue (Supplementary Fig. 3j, k). These results confirm that transplanted BMSCs can effectively repair bone defects after surviving under physiological oxygen concentrations and that hypoxia induces high rates of apoptosis of transplanted BMSCs, which severely limits the efficacy of BMSC transplantation.

**Fig. 7:**
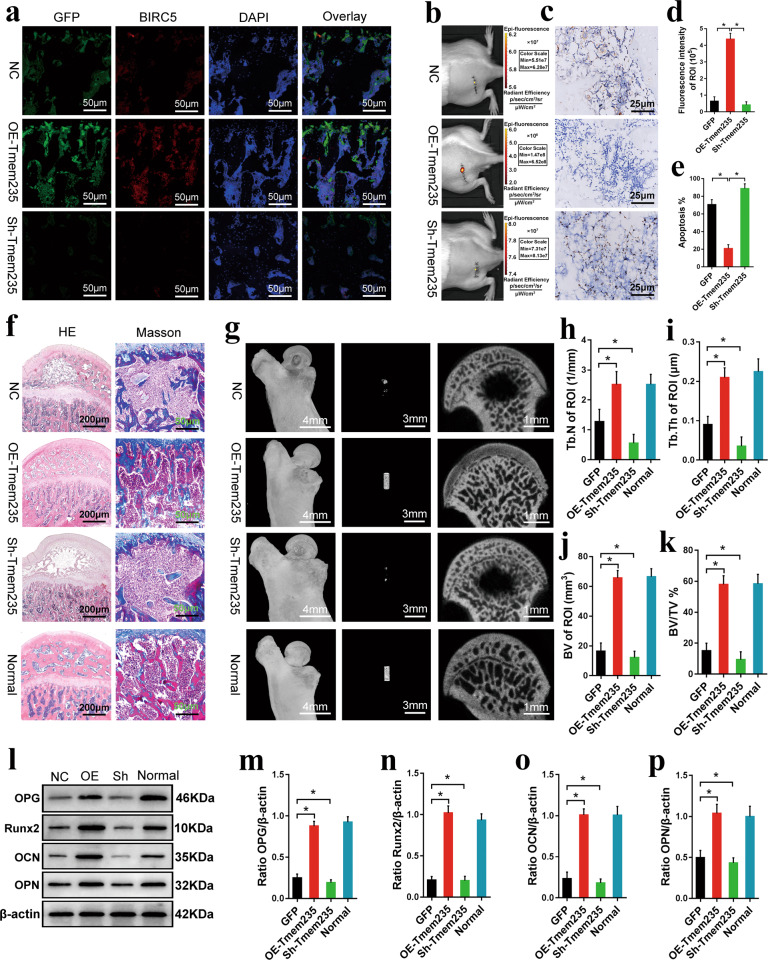
Lnc Tmem235 inhibits hypoxia-induced apoptosis of BMSCs and promotes repair of early SONFH. **a** At 2 days after operation, the expression levels of GFP and BIRC5 in the femoral head necrotic area were detected by immunofluorescence (*n* = 6); GFP green fluorescence labeled BMSCs, which could not penetrate bone tissue, green fluorescent protein (GFP), negative control (NC), overexpression (OE), short hairpin (Sh); **b** At 2 days after surgery, the fluorescence intensity of DiR in the transplantation area was detected by live imaging of small animals (*n* = 8); DiR red fluorescence labeled BMSCs, which can penetrate bone tissue; **c** At 2 days after surgery, TUNEL was used to detect apoptosis in the femoral head necrotic area (*n* = 6); **d** Quantitative analysis of DiR fluorescence intensity in the transplanted area as shown in **b** (*n* = 8); **e** Quantitative analysis of the proportion of TUNEL positive cells in the transplanted area as shown in **c** (*n* = 6); **f** At 12 weeks post-surgery, H&E staining and Masson staining were used to evaluate the repair of the necrotic area (*n* = 6); **g** At 12 weeks after surgery, micro-CT was used to analyze the repair of necrotic area of femoral head (*n* = 6); **h** Quantitative analysis of the number of trabeculae as shown in **g** (*n* = 6); **i** Quantitative analysis of the trabecular thickness as shown in **g** (*n* = 6); **j** Quantitative analysis of the volume of new bone tissue as shown in **g** (*n* = 6); **k** Quantitative analysis of the volume fraction of new bone tissue as shown in **g** (*n* = 6); **l**. At 12 weeks post-surgery, the levels of osteogenic markers (e.g., Runx2, OPN, OCN, and OPG) were detected by western blotting *(n* = 6); osteopontin (OPN); **m** Quantitative analysis of OPG expression as shown in **l** (*n* = 6); **n** Quantitative analysis of Runx2 expression as shown in **l** (*n* = 6); **o** Quantitative analysis of OCN expression as shown in **l** (*n* = 6); **p** Quantitative analysis of OPN expression as shown in **l** (*n* = 6). In (**d**, **e**, **h**–**k**, **m**–**p**), the data are normally distributed, and the variance is homogeneous. Data are presented as the means ± SDs; differences were tested using one-way ANOVA with Tukey’s *post-hoc* test; ^*^P < 0.05.

Our in vitro studies confirmed that Lnc Tmem235 inhibited hypoxia-induced apoptosis of BMSCs. Thus, we investigated whether Lnc Tmem235-mediated inhibition of hypoxia-induced apoptosis of BMSCs could improve the therapeutic effect of BMSCs on early SONFH. Lv-Lnc Tmem235 or Lv-Sh-Lnc Tmem235 was transfected into BMSCs to overexpress or silence Lnc Tmem235. Then, BMSCs were labeled with DiR and cocultured with XACB to construct tissue-engineered bone, which was transplanted to repair the early SONFH model. At 2 days post-surgery, we evaluated whether Lnc Tmem235 could inhibit the apoptosis of transplanted BMSCs in the osteonecrotic area by the fluorescence intensity of DiR, the expression levels of GFP and BIRC5, and the proportion of TUNEL-positive cells. The results showed that compared with the BMSCs/NC group, the Lnc Tmem235 overexpression group had higher DiR fluorescence intensity (Fig. 7b, d) and GFP and BIRC5 expression levels (Fig. 7a), and the proportion of TUNEL-positive cells was significantly decreased (Fig. 7c, e), while the Lnc Tmem235 low-expression group had lower DiR fluorescence intensity (Fig. 7b, d) and GFP and BIRC5 expression levels (Fig. 7a), and the proportion of TUNEL-positive cells was increased (Fig. 7c, e). These results suggest that Lnc Tmem235 inhibited apoptosis of BMSCs in the hypoxic microenvironment of osteonecrosis of the femoral head and promoted BMSC survival. At 12 weeks post-surgery, we evaluated the transplantation effect of BMSCs on early SONFH by micro-CT examination, HE staining, Masson staining, and the level of osteogenic markers. The results showed that compared with that of the BMSC/NC group, the defect area in the Lnc Tmem235 overexpression group was completely repaired (Fig. 7g), and the bone tissue tended to mature (Fig. 7f). The Tb.N (Fig. 7h), Tb.Th (Fig. 7i), BV (Fig. 7j), BVF (Fig. 7k), and the levels of osteogenic markers (e.g., Runx2, OPN, OCN, OPG) were significantly increased (Fig. 7l–p). Conversely, there was no obvious repair in the defect area in the Lnc Tmem235 low-expression group (Fig. 7f, g). The Tb.N (Fig. 7h), Tb.Th (Fig. 7i), BV (Fig. 7j), BVF (Fig. 7k), and levels of osteogenic markers (e.g., Runx2, OPN, OCN, and OPG) in the Lnc Tmem235 low-expression group were significantly lower than those in the BMSC/NC group (*P* < 0.05) (Fig. 7l–p). These results suggest that Lnc Tmem235 improved the therapeutic effect of BMSCs on early SONFH by inhibiting hypoxia-induced apoptosis of BMSCs.

## Discussion

BMSC transplantation has been shown to assist in repairing early SONFH. However, due to the hypoxic microenvironment in the necrotic area of the femoral head, transplanted BMSCs exhibit high rates of apoptosis, which seriously limits the osteogenic repair effect of BMSCs^38,39^. Therefore, methods that inhibit hypoxia-induced apoptosis of BMSCs are key to further improving the efficacy of BMSC transplantation. Recent studies have shown that lncRNAs regulate gene expression at many levels, such as at the transcriptional and post-translational levels, and participate in various physiological activities^40^. In the present study, we elucidated a key role of Lnc Tmem235 in reducing hypoxia-induced apoptosis of BMSCs. Specifically, we found that Lnc Tmem235 can act as a ceRNA to competitively bind to miR-34a-3p with BIRC5 mRNA to relieve the silencing effect of miR-34a-3p on BIRC5 mRNA, thus inhibiting hypoxia-induced apoptosis of BMSCs by promoting the expression of BIRC5. Additionally, we found that Lnc Tmem235 inhibition of hypoxia-induced apoptosis of BMSCs effectively improved the transplantation effect of BMSCs in treating early SONFH.

Hypoxia-induced apoptosis is one of the reasons for the limited efficacy of BMSC transplantation and is a major obstacle to clinical applications^41^. Although improving mitochondrial function, clearing ROS and interfering with apoptosis-related proteins can partially inhibit hypoxia-induced apoptosis of BMSCs, these effects remain unsatisfactory^18–21^. Therefore, it is important to develop new methods for improving the effects of BMSC transplantation. In recent years, with the continuous development of sequencing technology, the biological functions of lncRNAs have been continuously explored, and studies have shown that lncRNAs play an important role in regulating apoptosis. For example, Lnc ZFAS1 can inhibit apoptosis induced by calcium overload^22^. Moreover, lncRNAs are involved in the regulation of stress responses, such as hypoxia. For example, Lnc P21 can form a positive-feedback loop with HIF-1α, promoting hypoxia-related glycolysis in tumor cells^42^. However, the regulatory role of lncRNAs in the process of hypoxia-induced apoptosis remains poorly understood. In the present study, we screened hypoxia-reactive Lnc Tmem235 in a BMSC hypoxic model by lncRNA microarrays. Under hypoxic conditions, the expression of Lnc Tmem235 was downregulated, and its expression continued to be downregulated with the aggravation of hypoxia and an increased apoptotic rate, suggesting that Lnc Tmem235 may be related to hypoxia-induced apoptosis of BMSCs. Subsequently, we conducted functional experiments on Lnc Tmem235, and the results confirmed that Lnc Tmem235 could inhibit the hypoxia-induced apoptosis of BMSCs. These results indicate that lncRNAs regulate hypoxia-induced apoptosis, which provides novel targets for inhibiting hypoxia-induced apoptosis of BMSCs.

LncRNAs participate in various physiological activities mainly by regulating gene expression^43^. In our present study, we investigated the mechanism by which Lnc Tmem235 inhibited hypoxia-induced apoptosis of BMSCs. Under hypoxia, we analyzed the gene expression profile of BMSCs-Lnc Tmem235 by microarray and found that the expression of BIRC5 was significantly upregulated. Then, we verified the regulatory relationship between Lnc Tmem235 and BIRC5 in a BMSC hypoxic model. Specifically, we found that Lnc Tmem235 promoted the expression of BIRC5. BIRC5 is a member of the apoptosis inhibitor family, which can block apoptosis by inhibiting caspase activity^33–36^. To study the role of BIRC5 in inhibiting hypoxia-induced apoptosis of BMSCs by Lnc Tmem235, we silenced BIRC5 while upregulating Lnc Tmem235, which significantly weakened the antiapoptotic effect of Lnc Tmem235. Alternatively, we also upregulated BIRC5 while downregulating Lnc Tmem235, which mitigated hypoxia-induced apoptosis of BMSCs. These results indicated that Lnc Tmem235 inhibited hypoxia-induced apoptosis of BMSCs by regulating the expression of BIRC5.

LncRNAs can regulate gene expression through a variety of mechanisms, including pretranscriptional regulation (e.g., histone modification and DNA methylation), transcriptional regulation (e.g., enhancer activity, transcriptional interference, and control of transcription factors) and post-transcriptional regulation (e.g., regulation of alternative splicing, regulation of RNA subcellular location, regulation of RNA stability)^44–46^. Notably, increasing evidence has shown that lncRNAs can be used as ceRNAs to regulate the expression of target genes through miRNAs. For example, Lnc RoR can be used as a ceRNA to regulate the expression of Oct4, Nanog, and Sox2, which then interferes with the self-renewal of human embryonic stem cells^47^. SPAG5-AS1 can be used as a ceRNA to regulate the expression of SPAG5 to inhibit autophagy and apoptosis through the AKT/mTOR pathway^48^. In our present study, our bioinformatic predictions showed that Lnc Tmem235 and BIRC5 mRNA have the same binding site on miR-34a-3p and that Lnc Tmem235 and BIRC5 mRNA satisfy conditions for competitive binding to miR-34a-3p. Subsequently, our results confirmed that Lnc Tmem235 could be used as a ceRNA to bind miR-34a-3p competitively with BIRC5 mRNA to relieve the silencing effect of miR-34a-3p on BIRC5 mRNA and thereby regulate the expression of BIRC5. Collectively, these findings reveal a mechanism of Lnc Tmem235 as a ceRNA regulating gene expression from the level of post-transcriptional regulation.

In our present study, we used BMSCs to repair an early SONFH model, but the transplantation effect was not satisfactory. The main reason for this result was that a large number of BMSCs were apoptotic in the hypoxic microenvironment in the femoral head necrotic area, which seriously limited the transplantation effect of BMSCs on early SONFH. Strikingly, our in vitro results confirmed that Lnc Tmem235 inhibited hypoxia-induced apoptosis of BMSCs. Furthermore, to evaluate the effect of Lnc Tmem235 in vivo, we transplanted BMSCs overexpressing Lnc Tmem235 to repair the early SONFH model. The results showed that Lnc Tmem235-mediated inhibition of hypoxia-induced apoptosis of BMSCs effectively improved the therapeutic efficacy of BMSC transplantation.

**Fig. 8:**
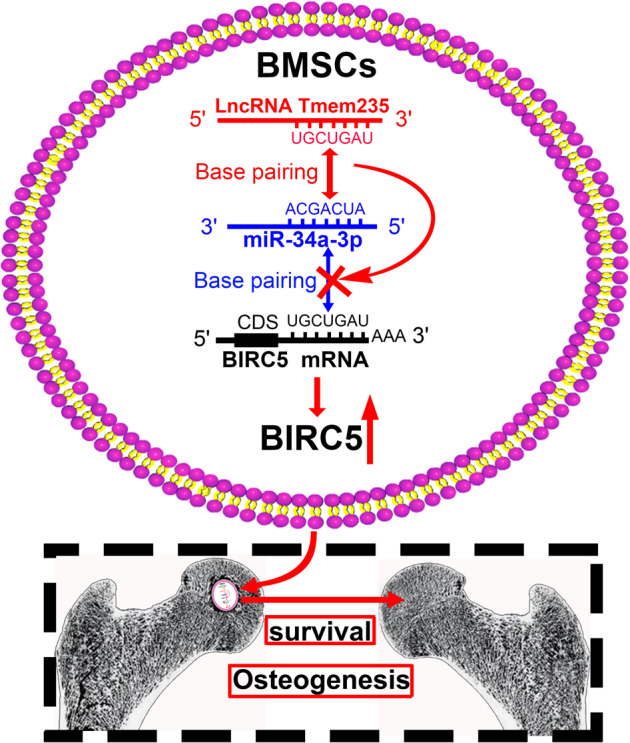
Role and mechanism of Lnc Tmem235 in repairing early SONFH by BMSC transplantation. Lnc Tmem235 competes with BIRC5 mRNA to bind miR-34a-3p, thereby releasing the silencing effect of miR-34a-3p on BIRC5 mRNA and promoting the expression of BIRC5 protein; this inhibits hypoxia-induced apoptosis of BMSCs in the femoral head necrotic area to ultimately improve the osteogenic repair effect of BMSCs on early SONFH.

In summary, we showed that Lnc Tmem235 inhibited hypoxia-induced apoptosis in BMSCs by regulating miR-34a-3p/BIRC5 and that Lnc Tmem235-mediated inhibition of hypoxia-induced apoptosis in BMSCs effectively improved the transplantation effect of BMSCs on early SONFH (Fig. 8). Collectively, our findings provide a new target and method for reducing hypoxia-induced apoptosis of BMSCs and improving the efficacy of BMSC transplantation.

## Supplementary information

supplementary materials

supplementary Figure 1

supplementary Figure 2

supplementary Figure 3

## Data Availability

Additional data or reagents are available from the corresponding author upon reasonable request.
