# Supplementary material for: Lnc Tmem235 promotes repair of early steroid-induced osteonecrosis of the femoral head by inhibiting hypoxia-induced apoptosis of BMSCs
Source: Exp Mol Med. 2022 Nov 16;54(11):1991–2006. doi: 10.1038/s12276-022-00875-0 (PMC9723185; doi:10.1038/s12276-022-00875-0)
Supplement: Supplementary file 1 — supplementary materials [file 12276_2022_875_MOESM1_ESM.doc]

**Supplementary materials:**

**1. Isolation, culture, and identification of BMSCs**

BMSC cell shape was that of a long spindle, and their growth pattern was fishlike (Supplementary Fig. 1a). After osteogenic induction, calcium nodules were formed, and the results of Alizarin Red and alkaline phosphatase (ALP) staining were positive (Supplementary Fig. 1b–c). After adipogenic induction, Oil Red O staining results were positive (Supplementary Fig. 1d). After chondrogenic induction, Alisin Blue staining results were positive (Supplementary Fig. 1e). In terms of surface antigens, positivity rates for Clusters of Differentiation 29, 90 and 106 (CD29, CD90 and CD106) were >99%, while those for CD11b and CD45 were respectively 5.82 ± 1.57% and 3.49 ± 1.43% (Supplementary Fig. 1f). Based on all of the above results, the BMSCs had typical morphological characteristics, growth patterns, surface markers, and multi-directional differentiation potential.

**Supplementary Fig. 1**. Isolation, culture, and identification of BMSCs: **a**. Primary BMSCs; **b**. Alkaline phosphatase staining after osteogenic differentiation of BMSCs (n = 3); **c**. Alizarin-red staining after osteogenic differentiation of BMSCs (n = 3); **d**. Oil-red-O staining after adipogenic differentiation of BMSCs (n = 3); **e**. Alisin-blue staining after chondrogenic differentiation of BMSCs (n = 3); **f**. Surface antigens of BMSCs—namely, CD11b, CD29, CD45, CD90, and CD106—were detected by flow cytometry (n = 5).

**2. Effects of different hypoxia conditions on BMSCs**

The oxygen concentration of BMSCs in the physiological environment of the bone marrow cavity is approximately 5%–10%, but the oxygen concentration in the femoral head necrosis area is < 1%, which is significantly lower than that in the physiological environment. When BMSCs were transplanted to repair femoral head necrosis, numerous apoptotic transplanted BMSCs were observed within 48 h of surgery. Therefore, oxygen concentration gradients were set as 20%, 10%, 5%, 1%, and 0%, and the time of hypoxia-treated cells was set as 48 h to explore the effects of different hypoxia conditions on BMSCs. The results showed no significant differences in the mitochondrial membrane potential, ATP content, ROS level, and apoptosis rate of BMSCs in the environment with oxygen concentration of 5%–10% compared with 20% oxygen concentration (Supplementary Fig. 2a–e, h–l), while cell viability and osteogenic activity showed an upward trend (Supplementary Fig. 2f, g, m). When the oxygen concentration was 1%, the mitochondrial membrane potential and ATP content were decreased, the ROS content was increased, the osteogenic activity and cell viability were decreased (Supplementary Fig. 2a–g, m), apoptosis-related proteins, such as Cleaved-CASP9 and Cleaved-CASP3, were up-regulated, and apoptosis was increased, all of which were statistically significant (Supplementary Fig. 2h–l). When the oxygen concentration was further reduced to 0%, the mitochondrial membrane potential and ATP content were significantly decreased, the ROS content was significantly increased, the osteogenic activity and cell viability were further reduced (Supplementary Fig. 2a–g, m), the expression of Cleaved-CASP9 and Cleaved-CASP3 was increased, and apoptosis was increased, all of which were statistically significant (Supplementary Fig. 2h–l). These results show that an appropriate hypoxia environment has no significant effect on BMSCs, and may even improve cell viability and osteogenic activity. However, when the oxygen concentration is < 1%, cells suffer irreversible damage and apoptosis. Because the oxygen concentration in the necrotic area of the femoral head is < 1%, we used the oxygen concentration of < 1% to establish the hypoxia model of BMSCs in vitro.

**Supplementary Fig. 2**. Effects of different hypoxia conditions on BMSCs. **a and b**. Mitochondrial membrane potential was detected by JC-1 (n = 5). **c**. Detection of ATP content (n = 6). **d and e**. ROS content detected by DCFH-DA (n = 5). **f and g**. The osteogenic activity of BMSCs was detected by alkaline phosphatase staining (n = 5). **h and i**. Apoptosis was detected by flow cytometry (n = 5). **j–l**. The expression of Cleaved-CASP9 and Cleaved-CASP3 was detected by immunoblotting (n = 4). **m**. Cell viability was detected by CCK-8 (n = 4). In (**b, c**, **e**, **g**, **i**, **k–m**), data are presented as means ± standard deviations (SDs); statistical significance was calculated by one-way ANOVA with Tukey’s *post hoc* tests; ^*^*P* < 0.05, ^**^*P* < 0.01, ^***^*P* < 0.001.

**3. Repair effect of BMSCs on SONFH under hypoxia and non-hypoxia conditions**

We transplanted BMSCs into the osteonecrosis area of SONFH (hypoxic environment) and the bone defect area of the normal femoral head (physiological oxygen concentration), with SONFH as the negative control and the normal femoral head as the positive control. Forty-eight hours after surgery, the oxygen concentration in the transplanted area, the expression of HIF-1α, and the apoptosis of transplanted BMSCs were detected. The results showed that the oxygen concentration in the transplanted area of the Normal/BMSCs group was higher than 5% (Supplementary Fig. 3c), the expression level of HIF-1α was extremely low (Supplementary Fig. 3a and b), and the local area was at a physiological oxygen concentration. The oxygen concentration in the transplanted area of the SONFH/BMSCs group and the osteonecrotic area of the SONFH group was significantly lower than 1% (Supplementary Fig. 3c), the expression level of HIF-1α was significantly increased (Supplementary Fig. 3a and b), and the local area was hypoxic. Compared with the Normal/BMSC group, the apoptosis of the transplanted BMSCs in the SONFH/BMSCs group was significantly increased, and the apoptotic rate was > 80% (Supplementary Fig. 3d and e). Twelve weeks after surgery, the repair of bone necrosis and bone defect was evaluated. Compared to the SONFH group, the osteonecrotic area was not significantly repaired in the SONFH/BMSCs group, there were no significant differences in bone volume fraction (BVF), trabecular number (Tb.N), and trabecular thickness (Tb.Th) (Supplementary Fig. 3f–i), and HE and Masson’s staining showed no obvious new bone formation (Supplementary Fig. 3j and k). Compared with the SONFH/BMSCs group, the bone defects in the Normal/BMSCs group were completely repaired, BVF, Tb.N, and Tb.Th were significantly increased (Supplementary Fig. 3f–i), and HE and Masson’s staining showed that the bone defects were completely filled with new bone tissue (Supplementary Fig. 3j and k). These results confirm that transplanted BMSCs can repair bone defects well after surviving under physiological oxygen concentration, and that hypoxia induces high rates of apoptosis of transplanted BMSCs, which severely limits the efficacy of BMSCs transplantation.

**Supplementary Fig. 3**. Repair effect of BMSCs on SONFH under hypoxia and non-hypoxia conditions. **a and b**. Immunostaining level of HIF-1α (n = 6). **c**. Oxygen concentration in the osteonecrosis area and transplantation area (n = 6). **d and e**. Apoptosis was detected by TUNEL staining (n = 6). **f**. Quantitative analysis of BVF (n = 6). **g**. Quantitative analysis of Tb.N (n = 6). **h**. micro-CT was used to evaluate the repair of bone defect (n = 6). **i**. Quantitative analysis of Tb.Th (n = 6). **j and k**. HE and Masson’s staining were used to detect new bone formation (n = 6). In (**a**, **c**, **d**, **f, g**, **i**), data are presented as the means ± SDs; statistical significance was calculated by one-way ANOVA with Tukey’s *post hoc* tests; ^*^*P* < 0.05, ^**^*P* < 0.01, ^***^*P* < 0.001.
