# Supplementary figures and images for: Lnc Tmem235 promotes repair of early steroid-induced osteonecrosis of the femoral head by inhibiting hypoxia-induced apoptosis of BMSCs
Source: Exp Mol Med. 2022 Nov 16;54(11):1991–2006. doi: 10.1038/s12276-022-00875-0 (PMC9723185; doi:10.1038/s12276-022-00875-0)

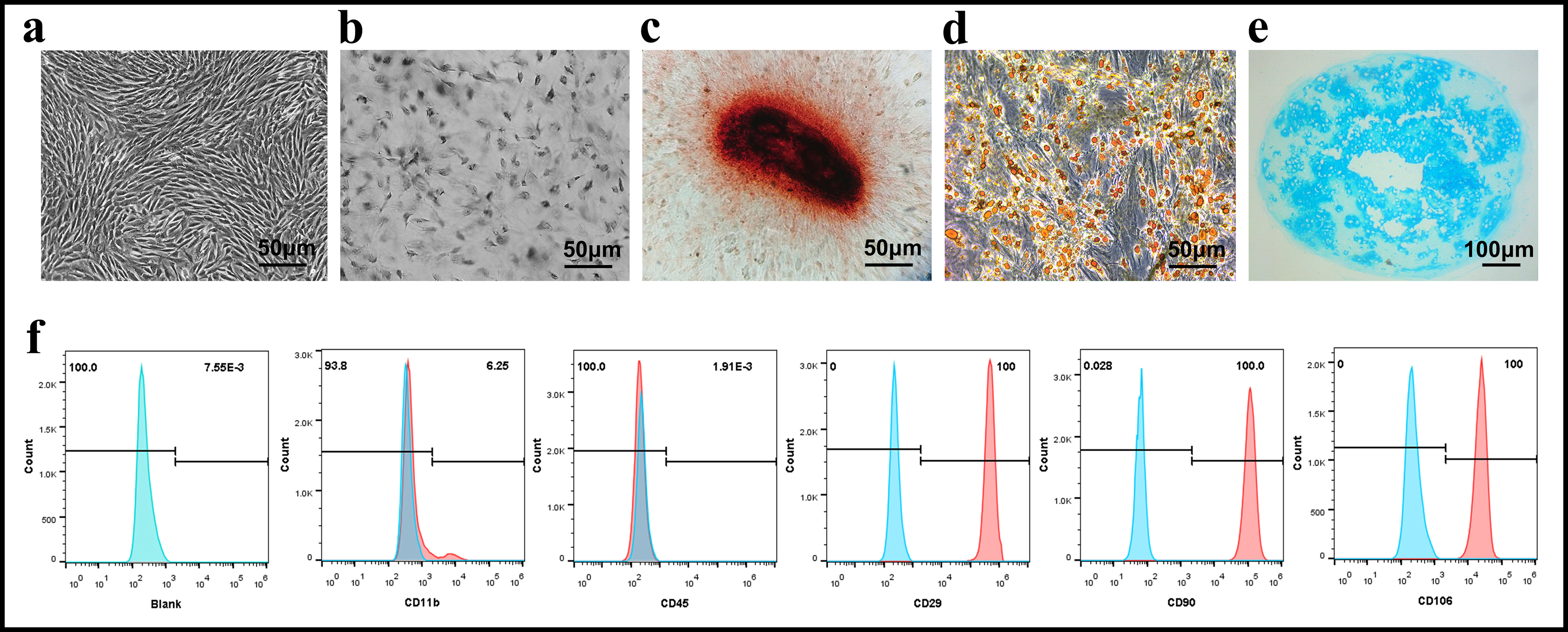

Supplement: Supplementary file 2 — supplementary Figure 1 [file 12276_2022_875_MOESM2_ESM.jpg]

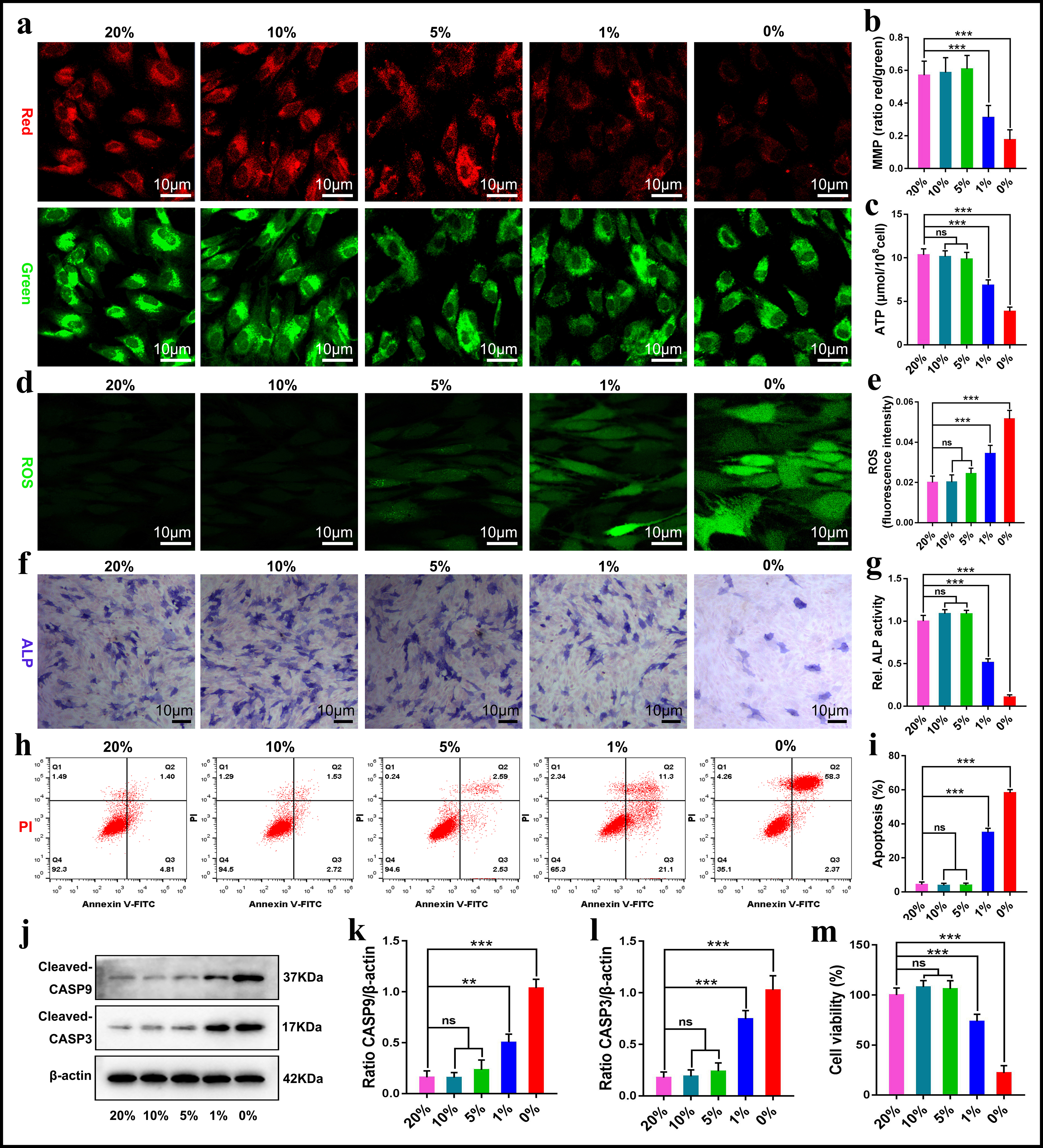

Supplement: Supplementary file 3 — supplementary Figure 2 [file 12276_2022_875_MOESM3_ESM.jpg]

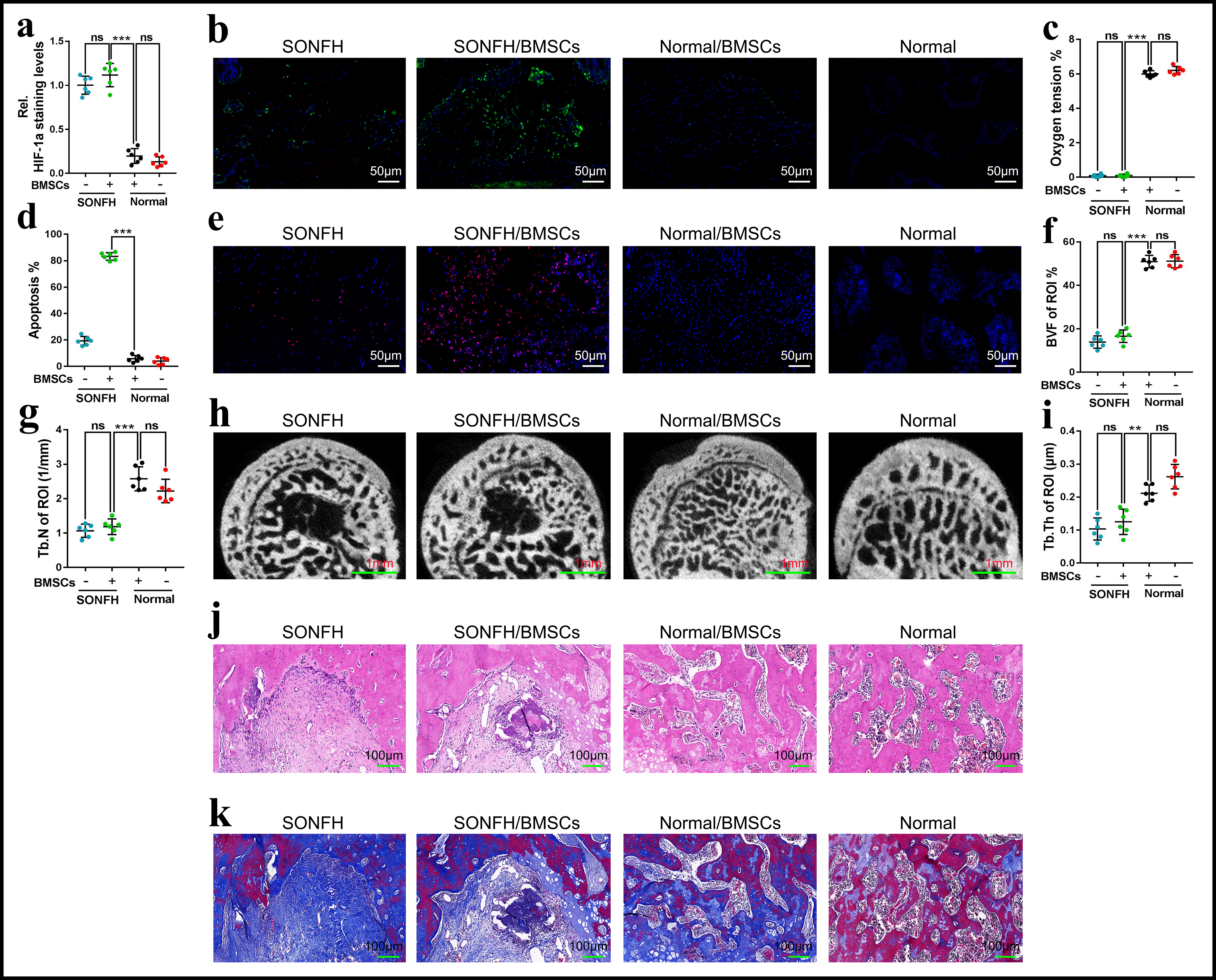

Supplement: Supplementary file 4 — supplementary Figure 3 [file 12276_2022_875_MOESM4_ESM.jpg]
